# SynAtlas: A Safe and Versatile Platform for Rapid Organ‐Scale Tissue Clearing and 3D Atlas Mapping

**DOI:** 10.1002/advs.78739

**Published:** 2026-11-09

**Authors:** Mei‐Xue Yuan, Xin‐Ya Qin, Ling‐Jie Li, Kai‐Lang Xiao, Meng‐Meng Zhang, Kun‐Qi Chen, Peng Chen, Yu Wang, Chen‐Wei Wang, Nan Zhou, Qing‐Hong Shan

**Affiliations:** ^1^ Institute of Brain Science The First Affiliated Hospital of Anhui Medical University Hefei China; ^2^ Department of Anatomy School of Basic Medical Sciences Anhui Medical University Hefei China; ^3^ Department of Neurosurgery Huashan Hospital, Shanghai Medical College Fudan University Shanghai China

**Keywords:** 3D mapping, aqueous clearing systems, orthogonal synergy, tissue clearing

## Abstract

Understanding the detailed three‐dimensional organization of cells, molecules, and tissues in their natural state is essential for advancing knowledge of biology, disease, and neural circuits, yet traditional slicing methods are slow, destructive, and prone to artifacts, while existing tissue‐clearing techniques often compromise speed, safety, signal preservation, or simplicity. By systematically analyzing the distinct optical barriers that impede tissue transparency, we propose an orthogonal synergy design principle. Guided by this framework, we developed SynAtlas (***Syn*** *ergistic* ***A*** *queous* ***T*** *issue c* ***l*** *e* ***a*** *ring* ***S*** *ystem*), a rationally designed clearing method that combines safe, compatible chemicals to simultaneously overcome the main barriers to transparency—lipid scattering, protein hydration heterogeneity, and refractive index mismatch—creating a fast, modular, and low‐hazard platform. SynAtlas clears thin sections in minutes and whole organs within two days while excellently preserving fluorescent proteins, nucleic acids, and antibody labels; it enables high‐resolution 3D atlases of neurons, vasculature, gene expression patterns, and cell types in mouse organs and archival human brain tissue, supports multi‐round labeling with minimal distortion, and allows non‐destructive cryo‐preservation for long‐term storage. This versatile approach makes advanced volumetric imaging more accessible and impactful for research and clinical applications.

## Graphical abstract

An orthogonal synergy design principle integrates lipid solubilization, protein hydration modulation, and refractive‐index matching within a unified aqueous clearing system. This modular strategy enables rapid, low‐hazard, and broadly compatible tissue clearing while preserving molecular signals, supporting multiscale three‐dimensional imaging across diverse organs and biological applications.

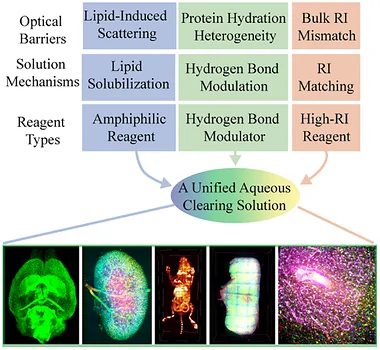

## 1 Introduction

High‐resolution three‐dimensional (3D) mapping of cells, molecules, and tissues in their native spatial context is essential for understanding complex biological systems, including neural circuits, organ function, disease pathology, and spatial heterogeneity in tumor microenvironments [1, 2]. Traditional histological sectioning, while valuable, is labor‐intensive, prone to deformation, material loss, and sample destruction, limiting scalable volumetric analysis [3]. Optical tissue clearing, combined with light‐sheet or advanced fluorescence microscopy, has enabled non‐destructive deep imaging of intact organs and organisms by minimizing light scattering [4].

Tissue‐clearing methods are broadly classified into solvent‐based (hydrophobic), aqueous‐based (hydrophilic), and hydrogel‐embedding approaches [5, 6]. Solvent‐based techniques (e.g., 3DISCO, iDISCO, PEGASOS, FDISCO) achieve rapid and efficient transparency in large‐volume samples through dehydration and high‐refractive‐index (RI) organic solvents [7, 8, 9, 10], but they often involve multi‐step dehydration, tissue shrinkage, and toxic reagents that pose environmental and safety risks. Aqueous‐based methods (e.g., CUBIC series, ScaleS, SeeDB) offer superior biocompatibility, excellent preservation of fluorescence signals and tissue morphology [11, 12, 13], yet typically require prolonged incubation times—even optimized variants like CUBIC‐R+ [14]. Hydrogel‐based variants (e.g., CLARITY, PACT, SHIELD) enhance mechanical and chemical stability via hydrogel embedding, enabling multi‐round labeling of proteins and RNA [15, 16, 17], but demand specialized expertise and extended multi‐step workflows.

Recent innovations have accelerated hydrophilic clearing and improved preservation, including EZ Clear, OptiMuS, LIMPID, and ADAPT‐3D [18, 19, 20, 21]. On the solvent side, SOLID minimizes distortion for accurate 3D registration and analysis [22]. Meanwhile, VIVIT introduces ionic liquids to achieve glassy‐state clearing with minimal distortion, high transparency, and enhanced low‐temperature preservation [23]. However, these advances primarily optimize specific aspects (e.g., speed, distortion, or compatibility) rather than integrating all core requirements within a unified system. Consequently, there remains a critical gap for a comprehensive method that simultaneously delivers broad compatibility, rapid processing, simple workflows, low‐toxicity biosafety, and potential for bio‐banking. These persistent trade‐offs limit the widespread adoption of tissue clearing in standard laboratories and clinical settings, where fast, safe, user‐friendly, and broadly applicable protocols are essential for high‐throughput studies, multi‐modal phenotyping, and translational applications involving valuable human or archival samples [4, 6].

To overcome these limitations, we moved beyond empirical trial‐and‐error and adopted a rational design strategy termed “orthogonal synergy.” We hypothesized that the three primary barriers to optical transparency—lipid‐induced scattering, protein hydration heterogeneity, and bulk RI mismatch—represent orthogonal optical obstacles arising from distinct physical origins [6, 24]. Therefore, we reasoned that these barriers could be resolved through a chemically unified and synergistic strategy. Crucially, we conceived this strategy not as a fixed formulation, but as an open, modular framework. While the core triad targets the three universal scattering sources, the system's chemical compatibility allows for the integration of auxiliary modules—such as decolorization agents for heme‐rich tissues [11, 14, 25]—to overcome tissue‐specific absorption barriers without disrupting the central synergy. By deploying distinct, chemically compatible modules that target each specific barrier within a coherent framework, we aimed to eliminate the chemical antagonism often found in multi‐step protocols (e.g., incompatibility between delipidation and RI matching agents). Guided by this principle, we developed SynAtlas as a direct implementation of this framework. The core formulation relies on the synergy of three functionally distinct components: an amphiphilic agent for lipid solubilization, a hydrogen‐bond modulator for protein hydration, and a high‐RI reagent for bulk refractive index homogenization [14]. Because this architecture aligns specific chemical modules with these discrete optical barriers, it provides a robust and generalizable design principle. Such targeted modularity enables rational selection and optimization of reagent combinations, substantially reducing the screening burden while allowing precise engineering of clearing agents with tailored properties.

Here, by rationally substituting distinct chemical modules to design various tissue‐clearing variants, we demonstrate the exceptional flexibility of the orthogonal synergy principle in guiding formulation development. Built upon this framework, we demonstrate that SynAtlas provides a powerful, unified platform for multi‐scale and multi‐modal 3D biological mapping. The protocol achieves rapid transparency—clearing thin tissue sections within minutes, whole organs within two days, and juvenile mice (including intact bone) within three days—while maintaining an excellent, low‐toxicity safety profile suitable for routine laboratory use. Crucially, validating our modular design principle, we show that the core components of SynAtlas can be rationally substituted with alternative chemical modules to tailor secondary properties without compromising clearing velocity. We demonstrate the broad multi‐modal applicability of the SynAtlas platform through diverse, high‐throughput applications: constructing high‐resolution 3D atlases of whole‐organ neuronal and vascular networks, performing multi‐round multiplexed RNA fluorescence in situ hybridization (FISH) at single‐cell resolution, and enabling multiplexed, multi‐round immunostaining in archival human cortical tissues despite prolonged fixation. Collectively, this orthogonal‐synergy‐guided approach not only delivers a robust, biobanking‐compatible tool for volumetric imaging but also establishes a conceptual framework for the rational design of next‐generation tissue‐clearing chemistry.

## 2 Results

### 2.1 Rational Design and Physicochemical Implementation of the Orthogonal Synergy Framework

To translate the “orthogonal synergy” hypothesis into a functional platform, unlike conventional tissue‐clearing methods that typically employ a sequential approach to address individual optical barriers step‐by‐step, we sought to concurrently address the three primary optical barriers—lipid scattering, protein hydration heterogeneity, and bulk RI mismatch—through a unified chemical framework (Figure 1, A). Guided by this principle, we performed a systematic screen of over 1600 reagents from the CUBIC library [14], narrowing them down to 293 low‐hazard candidates (Figure S1 A). Following functional classification according to the three optical barriers, representative candidates from each module were experimentally combined and evaluated for clearing performance, ultimately identifying urea, 1,2‐hexanediol, and iohexol as the optimal synergistic triad, and formulating SynAtlas (10% (v/v) 1,2‐hexanediol, 24% (w/v) urea, and 60% (w/v) iohexol; Table S1 and Figure S1A–D). In this rationally designed architecture, 1,2‐hexanediol acts as a hydrotropic amphiphile to disrupt lipid packing; urea modulates the hydrogen‐bond network to solvate hydrophobic protein domains; and iohexol provides a high‐RI environment. We validated this synergy by testing component combinations. While individual reagents or binary mixtures (e.g., 10% (v/v) 1,2‐hexanediol + 60% (w/v) iohexol) only partially improved tissue transmittance, optimal clearing efficacy was achieved only when all three functional modules were combined, confirming the necessity of the orthogonal synergy design (Figure 1B,C).

**FIGURE 1:**
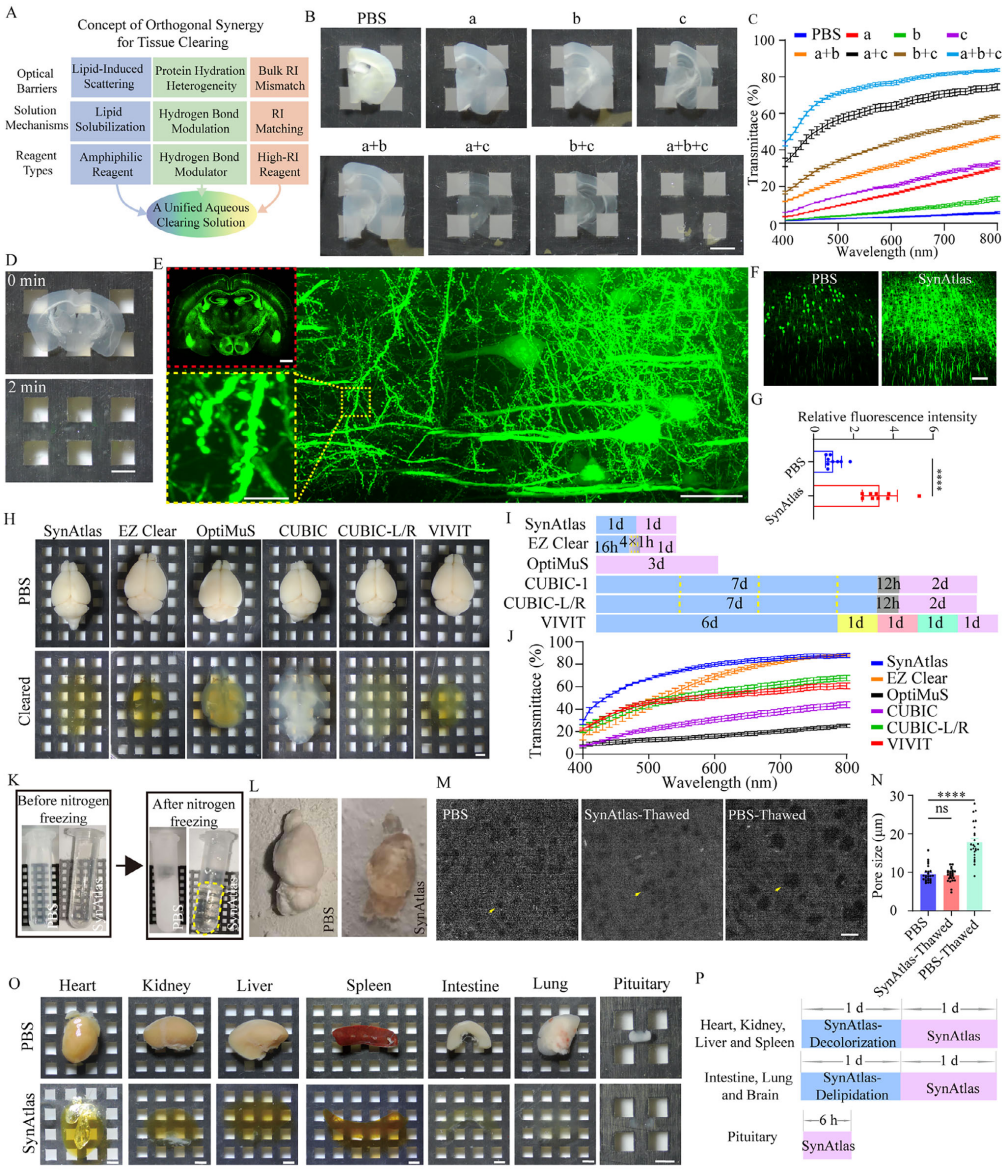
Development and optimization of SynAtlas (A) Concept of orthogonal synergy for tissue clearing. (B) Representative images showing 300‐µm mouse brain sections after 10‐min treatment with individual reagents or reagent combinations. a: 10% (v/v) 1,2‐hexanediol; b: 24% (w/v) urea; c: 60% (w/v) iohexol. Scale bar, 2.5 mm. (C) Optical transmittance spectra of 300‐µm mouse brain sections after the indicated treatments, measured over the wavelength range of 400–800 nm. (*n* = 3 per group). (D) Representative images of 100‐µm Thy1‐EYFP brain sections before and after 2‐min SynAtlas treatment. Scale bar, 2.5 mm. (E) Representative fluorescence images of 100‐µm Thy1‐EYFP mouse brain sections cleared with SynAtlas. The upper‐left inset shows a whole‐section fluorescence image (scale bar, 1 mm; region outlined by red dashed box). The lower‐left inset shows a magnified view of the region indicated by the yellow dashed box in the main panel, revealing detailed neuronal morphology, including spine structures (scale bar, 4 µm). The main panel displays neuronal somata, dendrites, and spines within the cleared cortical tissue (scale bar, 50 µm). (F) Representative fluorescence images of 100‐µm Thy1‐EYFP mouse brain sections in PBS and after SynAtlas treatment. Scale bar, 100 µm. (G) Quantification of YFP fluorescence intensity in PBS and SynAtlas (*n* = 9 for each group). (H) Representative whole‐brain clearing results obtained with SynAtlas and other established methods. Scale bars, 2.5 mm. (I) Comparison of different clearing methods in terms of processing time and the number of operational steps, with yellow dashed lines indicating solution‐refreshing steps, corresponding to the whole‐brain clearing results shown in (H). (J) Optical transmittance spectra recorded across the 400–800 nm wavelength range for tissues cleared using the indicated methods. (*n* = 3 per group). (K) Comparison of PBS and SynAtlas solutions before and after liquid‐nitrogen freezing. (L) Representative images of PBS‐immersed and SynAtlas‐cleared mouse half‐brains after rapid freezing in liquid nitrogen, showing maintained transparency in SynAtlas‐treated samples. (M) Representative images of brain tissues from PBS control, PBS freeze–thaw, and SynAtlas freeze–thaw groups, illustrating suppression of ice crystal–induced pore formation after SynAtlas treatment. Scale bars, 20 µm. (N) Quantification of cryo‐induced pore diameters in tissues before and after freeze–thaw cycles, demonstrating suppression of ice crystal formation in SynAtlas‐treated samples (*n* = 25 for each group). (O) Application of SynAtlas to clear heme‐rich and other mouse organs. Scale bars, 2.5 mm. (P) Flowchart illustrating the transparency clearing process for different tissues shown in (O).

SynAtlas rendered 100‐µm Thy1‐EYFP brain sections transparent within 2 min while enhancing YFP fluorescence intensity more than threefold relative to PBS (Figure 1D–G) and preserving fine dendritic structures (Figure 1E). Thicker sections (500, 1000, and 2000 µm) were rendered transparent via one‐step immersion within 1, 5, and 24 h, respectively (Figure S2A). The linear expansion of 2000‐µm sections after 24 h was not statistically significant (P = 0.1523), indicating minimal tissue deformation (Figure S2B).

### 2.2 Scalability, Cryo‐Stability, and Validation of the Orthogonal Synergy Framework

We next scaled the SynAtlas framework to intact whole organs. For whole mouse brains, direct immersion in the high‐osmolarity SynAtlas solution caused tissue contraction and limited penetration (Figure S2C). To address this, we implemented a stepwise equilibration strategy: tissues were first equilibrated in a low‐osmotic delipidating solution (SynAtlas‐Delipidation)—which lacks the high‐concentration RI‐matching agent—for 1 day, followed by incubation in SynAtlas for 1 day for final clearing. This workflow rendered whole brains uniformly transparent within 2 days (Figure S2D). Benchmarking against established methods (EZ Clear, OptiMuS, CUBIC, CUBIC‐L/R and VIVIT) revealed that SynAtlas and EZ Clear achieve comparable or superior transparency within 2 days—substantially faster than the approximately 10‐day protocols required by multi‐step alternatives (Figure 1H,I). Furthermore, quantitative transmittance analysis demonstrated that SynAtlas consistently achieved superior light transmittance across a broad spectrum of wavelengths compared to the evaluated benchmarks (Figure 1J).

We next evaluated RNA preservation using propidium iodide (PI) staining to label RNA. PI fluorescence intensity in SynAtlas‐cleared tissues was comparable to PBS‐treated controls, whereas all other clearing methods caused significant signal loss (Figure S3A,B). Morphologically, PBS, SynAtlas, and EZ Clear preserved intact somatic outlines. In contrast, the other methods yielded PI signals that almost completely colocalized with DAPI, indicating cytoplasmic RNA degradation. Consistent with these findings, fluorescence in situ hybridization (FISH) revealed robust mRNA signals only in SynAtlas‐, EZ Clear‐, and PBS‐treated tissues; no FISH signal was detected in OptiMuS, CUBIC, CUBIC‐L/R, or VIVIT groups, likely due to RNA degradation during extended processing (Figure S3C). To evaluate antigen preservation and immunostaining quality following tissue clearing, we performed immunofluorescence staining for NeuN and Iba1 and compared SynAtlas with several established tissue‐clearing methods (Figure S3D–J). For NeuN staining, SynAtlas significantly increased fluorescence signal intensity compared with PBS (Figure S3E), accompanied by an increase in background fluorescence (Figure S3F), resulting in an SNR comparable to that of PBS (Figure S3G). Among the other clearing methods, EZ Clear and OptiMuS exhibited significantly higher NeuN SNRs than PBS. For Iba1 staining, SynAtlas showed no significant differences from PBS in signal intensity, background fluorescence, or SNR (Figure S3H–J). Representative images showed well‐defined NeuN‐ and Iba1‐positive structures after SynAtlas treatment, comparable to those observed with the other clearing methods (Figure S3D). Collectively, these results indicate that SynAtlas preserves NeuN and Iba1 antigenicity while maintaining immunostaining quality and imaging SNR.

Cryopreservation is a standard practice for preserving structural integrity and stabilizing biomolecules such as RNA. While evaluating SynAtlas, we discovered a striking physical property under cryogenic conditions that distinctly differs from conventional aqueous buffers. While standard aqueous buffers (e.g., PBS) crystallize into opaque solids upon rapid cooling, SynAtlas transitions into a transparent, amorphous solid state without visible ice formation (Figure 1K). This apparent vitrification suggests that SynAtlas effectively suppresses water crystallization, a feature with significant implications for cryo‐preservation. To test the cryo‐stability of the cleared tissues, SynAtlas‐processed half brains were subjected to rapid freezing in liquid nitrogen. The tissues remained transparent in the frozen state (Figure 1L). Freeze–thaw analysis revealed that pore diameters in SynAtlas‐treated tissues were indistinguishable from non‐frozen PBS controls, whereas frozen–thawed PBS samples exhibited significant structural damage due to ice crystal formation (Figure 1M,N). This confirms that SynAtlas effectively suppresses ice crystallization at the cellular level. To further evaluate the efficacy of low‐temperature storage on biomolecule preservation, SynAtlas‐cleared hemispheres from Thy1‐YFP mice were stored at either 4°C or −80°C for two weeks (Figure S4A). We then assessed the preservation of endogenous fluorescence and RNA using light‐sheet microscopy and histological sectioning. Quantitative analysis revealed no significant differences in total YFP fluorescence intensity or sectional signal density between the 4°C and −80°C groups (Figure S4B–D). Similarly, PI staining intensities remained comparable between the two storage conditions (Figure S4E,F). Furthermore, tissues from both groups supported robust FISH labeling (Figure S4G), demonstrating that SynAtlas‐processed samples can be effectively stored at either 4°C or −80°C without compromising endogenous fluorescent proteins or nucleic acid biomarkers.

Having validated the efficacy of SynAtlas on tissue sections and whole brains, we next investigated the applicability of the orthogonal synergy framework to large‐volume samples. First, using urea as a representative hydrogen‐bond modulator, we tested its impact on clearing efficacy. Among the tested gradients (0, 6, 12, 18, and 24% (w/v)), only 24% (w/v) urea achieved complete transparency (Figure S5A,B). Crucially, since the bulk RI of these solutions is dominantly established by the constant 60% (w/v) iohexol, the failure of lower urea concentrations to clear tissue—despite possessing a similar RI—provides compelling evidence that bulk RI matching and lipid removal alone are insufficient. Instead, a critical concentration of a hydrogen‐bond modulator is required to synergistically modulate hydrogen bonding and solvate hydrophobic domains within the solution.

Furthermore, we evaluated the choice of different amphiphilic agents on the orthogonal synergy by comparing 1,2‐hexanediol against common detergents (SDS, SDBS, Tween‐80, and Triton X‐100). 1,2‐hexanediol demonstrated superior performance in overall transparency. In contrast, SDS and SDBS achieved good clearing only in the olfactory bulb and partial surface regions. The Triton X‐100 group exhibited relatively good overall transparency but was inferior to 1,2‐hexanediol, while the Tween‐80 group appeared uniformly white, failing to achieve sufficient transparency (Figure S5C,D). This superiority of 1,2‐hexanediol likely stems from a fundamental mechanistic difference: whereas traditional detergents rely on the formation of large micelles to sequester lipids—which can be sterically hindered in dense tissue matrices—1,2‐hexanediol functions through a hydrotropic mechanism. As a small molecule, it solubilizes lipids via direct molecular interaction without forming bulky aggregates, ensuring more rapid tissue permeation than micellar systems.

Additionally, we tested the effect of different high‐RI agents (e.g., antipyrine, uridine) on the orthogonal synergy (Figure S5E). Results showed that alongside iohexol, the antipyrine and uridine groups also exhibited good tissue clearing efficacy, whereas the 1‐(2‐Hydroxyethyl)‐piperazine group performed poorly (Figure S5F). Quantitative transmittance analysis further confirmed that the final SynAtlas formulation exhibited the highest overall transmittance across 400–800 nm among the representative formulations tested (Figure S5G). Furthermore, reagents like Nicotinamide and Creatinine could not be tested due to their insolubility in the current system (Figure S5H). These results indicate that while the orthogonal synergy principle guides the selection of functional modules, specific physicochemical compatibility (e.g., solubility and RI contribution) remains critical for formulation success.

To further validate the design logic under the orthogonal synergy principle for formulating rapid tissue‐clearing reagents, we substituted urea with N‐methyl‐D‐glucamine (NMDG), which has been reported to exhibit superior hydrogen‐bond modulation properties [26]. Formulations utilizing NMDG achieved rapid half‐brain transparency within 24 h (Figure S6A–C), confirming that the hydrogen‐bond modulator is a modular, interchangeable component. Beyond utilizing single high‐RI agents, we also evaluated high‐RI mixtures for RI matching. Specifically, we tested the ionic liquid combination of 1‐(3‐aminopropyl)imidazole (NAPID) and phthalic acid (PA) from the VIVIT protocol, as well as the antipyrine/nicotinamide combination from CUBIC‐R (Figure S6D,E). In the presence of 10% 1,2‐hexanediol and 24% urea or NMDG, the NAPID/PA formulations remained as clear solutions at room temperature (designated S1 and S2). Conversely, the antipyrine/nicotinamide combinations crystallized into solids under identical conditions (designated S3 and S4). However, when the NMDG concentration was reduced to 12%, the antipyrine/nicotinamide mixture formed a stable, clear liquid at room temperature (designated S5) (Figure S6F). We subsequently evaluated the rapid clearing efficacy of the stable liquid formulations (S1, S2, and S5) (Figure S6G). All three combinations successfully cleared mouse half brains within 24 h (Figure S6H). Finally, cryogenic exposure at −80°C revealed that while formulation S1 solidified into an opaque state, formulations S2 and S5 remained completely transparent (Figure S6I,J). Collectively, these results demonstrate that distinct modular combinations can yield novel, rapid clearing systems, with their secondary properties—such as cryopreservation compatibility—tightly coupled to the specific chemical components chosen.

To validate the modular extensibility of the SynAtlas framework for addressing specific optical barriers beyond light scattering, we integrated a decolorization module targeting hemoglobin‐rich organs. We evaluated the synergistic effects of several decolorizing agents, including Quadrol, N‐butyldiethanolamine, and N‐methyldiethanolamine, in combination with the SynAtlas‐Delipidation formulation. The results showed that N‐butyldiethanolamine exhibited the strongest synergistic effect (Figure S7A,B). Accordingly, N‐butyldiethanolamine was incorporated into the delipidation step, yielding the SynAtlas‐Decolorization protocol. This optimized protocol efficiently cleared a variety of organs, including the heart, kidney, liver, and spleen (Figure 1O). Furthermore, substituting N‐butyldiethanolamine with Quadrol or N‐methyldiethanolamine similarly achieved robust decolorization and optical transparency (Figure S7C,D). Given that these two alternative reagents carry no GHS “Danger” signal words and present lower hazard profiles, they serve as ideal low‐toxicity alternatives for the decolorization module. Collectively, these results demonstrate that the SynAtlas framework is highly scalable, cryo‐stable, and broadly applicable across diverse tissue types (Figure 1P).

### 2.3 3D Cell‐Type and Projection Atlases with Fluorescent Protein Labels

We next assessed the performance of SynAtlas in constructing three‐dimensional cell‐type and projection atlases using diverse fluorescent protein (FP) labels. Brains from Thy1‐EYFP (EYFP), CRH‐Cre;Ai9 (tdTomato), VIP‐Cre;Ai6 (ZsGreen), and ChAT‐Cre;Ai6 (ZsGreen) mice were cleared using SynAtlas. Endogenous FP signals were well preserved and remained bright after clearing (Figure 2A and Figure S8A–D).

**FIGURE 2:**
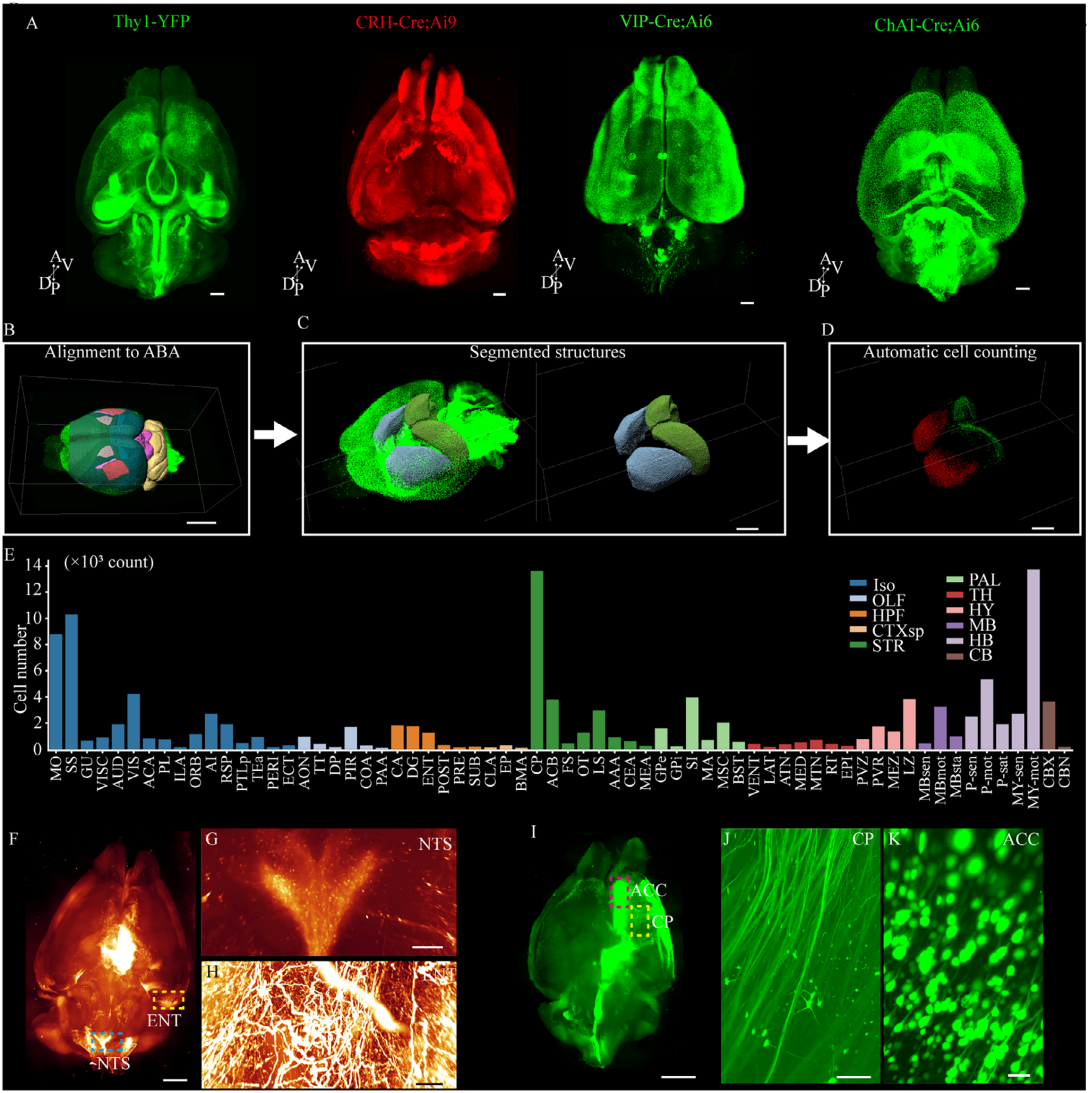
Whole‐brain 3D mapping of neuronal populations and projections enabled by SynAtlas clearing. (A) Whole‐brain 3D light‐sheet fluorescence imaging of Thy1‐EYFP, CRH‐Cre; Ai9, VIP‐Cre; Ai6, and ChAT‐Cre; Ai6 mice following SynAtlas clearing. Scale bars,1000 µm. (B) Whole‐brain reconstruction of ChAT‐Cre; Ai6 mice registered to the Allen Brain Atlas, enabling spatially resolved mapping of cholinergic neurons. Scale bar, 2000 µm. (C) Representative example showing extraction of hippocampus and striatum for regional analysis. Scale bar,1000 µm. (D) Example of automated cell detection and quantification in the hippocampus and striatum using the Imaris “Spot” function. Scale bar, 1000 µm. (E) Quantification of ChAT+ cell numbers across major brain regions, illustrating the region‐specific distribution pattern. (F) Whole‐brain 3D imaging of CRH‐Cre mice injected with AAV‐DIO‐mtdTomato in the PVN, following SynAtlas clearing. Scale bar, 2000 µm. (G) Magnified view of the dashed box region in (F), showing PVN CRH neuronal projections to the NTS. Scale bar, 400 µm. (H) Magnified view of the solid line‐outlined box region in (F), revealing dense and well‐defined PVN CRH neuronal fibers within the entorhinal cortex. Scale bar, 300 µm. (I) Whole‐brain 3D imaging of C57BL/6J mice injected with AAV/retro‐hSyn‐EGFP in the VTA, following SynAtlas clearing. Scale bar, 3000 µm. (J) Enlarged view of the boxed region in (I), highlighting striatal fibers projecting to the VTA. Scale bar, 200 µm. (K) Enlarged view of the boxed region in (I), showing retrogradely labeled ACC neurons projecting to the VTA. Scale bar, 100 µm.

In ChAT‐Cre;Ai6 mice, minimal morphological distortion enabled precise registration of cleared brains to the Allen Brain Atlas (ABA), followed by anatomical segmentation (Figure 2B–D; Figure S8E and movie S1). Automated cell detection in Imaris allowed quantification of Choline Acetyltransferase (ChAT) neurons across > 66 regions, including: Isocortex (Iso), olfactory area (OLF), hippocampal formation (HPF), Cortical subplate (CTXsp), Striatum (STR), Pallidum (PAL), Thalamus (TH), Hypothalamus (HY), Midbrain (MB), Hindbrain (HB), and Cerebellum (CB) (Figure 2E). The Caudoputamen (CP) contained the largest ChAT+ population, consistent with known distributions of intrinsic cholinergic neurons [27, 28].

We then combined SynAtlas with viral anterograde and retrograde tracing. For anterograde labeling, AAV‐DIO‐mtdTomato was injected into the paraventricular nucleus (PVN) of CRH‐Cre mice. After SynAtlas clearing, light‐sheet imaging revealed intact long‐range projections (Figure 2F and movie S2), including dense PVN projections to the nucleus tractus solitarius (NTS; Figure 2G) and fibers in the entorhinal cortex (ENT) (Figure 2H). For retrograde tracing, AAV/retro‐hSyn‐EGFP was injected into the ventral tegmental area (VTA). SynAtlas clearing allowed visualization of VTA‐projecting neurons and fibers, including inputs in the striatum and neuronal somata in the anterior cingulate cortex (ACC; Figure 2I–K and movie S3).

These results indicate that SynAtlas preserves diverse FP labels, supports accurate whole‐brain registration and quantitative cell‐type analysis, and enables comprehensive mapping of anterograde and retrograde projections for large‐scale circuit anatomy and connectomics.

### 2.4 3D Cellular and Vascular Mapping with Nucleic Acid and Vascular Labels

To extend SynAtlas to non‐genetic labels, we combined it with nucleic acid dyes and vascular tracers. PI was used for whole‐brain nucleic acid labeling in Thy1‐EYFP mice. After SynAtlas clearing, PI provided uniform 3D labeling throughout the brain while preserving Thy1‐EYFP signals, enabling single‐cell‐resolution imaging across the entire organ (Figure 3A). Optical sectioning confirmed consistent PI intensity in both cortical and deep regions, including the brainstem (Figure 3B,C and movie S4).

**FIGURE 3:**
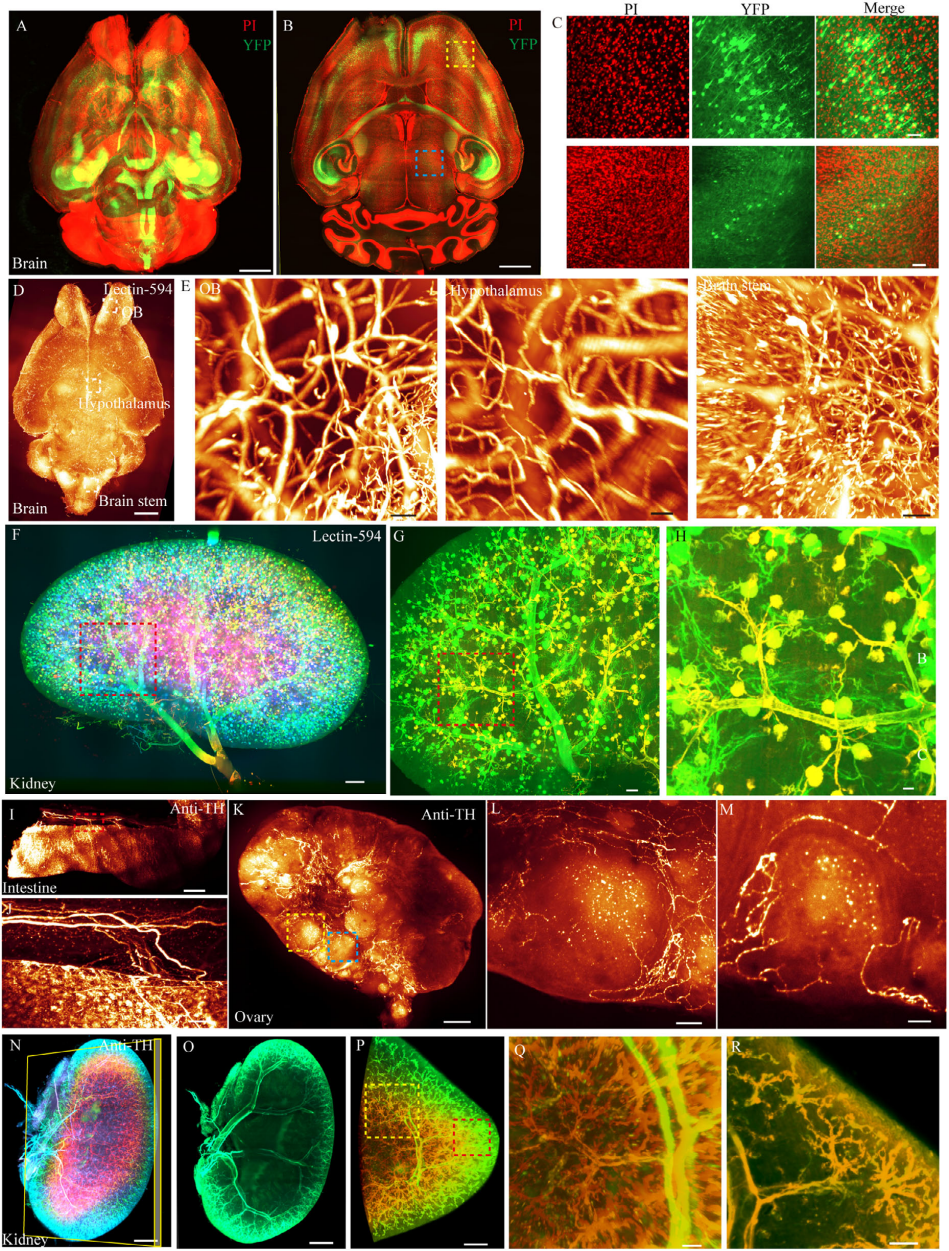
SynAtlas enables organ‐scale 3D mapping of cellular, vascular, and peripheral sympathetic architectures. (A) Whole‐brain 3D rendering of a Thy1‐EYFP mouse after PI staining, with YFP‐labeled neurons in green and PI‐labeled cells in red. Scale bar, 2 mm. (B) Representative horizontal sections extracted from the whole‐brain dataset shown in (A), displaying PI staining nuclei across multiple brain regions. Scale bar, 2 mm. (C) Magnified views of boxed regions in (B). Upper panel: cortical region in yellow box showing YFP‐ and PI‐labeled cells. Lower panel in blue box: brainstem region exhibiting YFP‐ and PI‐labeled cells. Scale bar, 150 µm. (D) Whole‐brain 3D reconstruction of the vascular network labeled with Lectin‐594. Scale bar, 2000 µm. (E) Enlarged views of boxed regions in (D), showing the labeled microvasculature in the OB, hypothalamus, and brainstem, with clear visualization of fine branching patterns. Scale bar, 100 µm. (F) 3D reconstruction of the kidney vasculature labeled with Lectin‐594 and color‐coded by depth. Scale bar, 700 µm. (G) Magnified view of the boxed region in (F), showing detailed renal vessels and branching patterns. Scale bar, 200 µm. (H) Further magnified view of the boxed region in (G), revealing fine microvasculature and glomerular structures with high spatial resolution. Scale bar, 100 µm. (I) 3D rendering of TH immunostaining in the small intestine, showing extensive sympathetic innervation. Scale bar,1000 µm. (J) Magnified view of the boxed region in (I), revealing dense TH+ fibers distributed within the muscular layer of the small intestine. Scale bar, 100 µm. (K) 3D reconstruction of TH immunostaining in the ovary. Scale bar, 300 µm. (L to M) Enlarged views of the boxed regions in (K), showing TH+ fibers surrounding the corpus luteum. Scale bars, 70 µm for (L); 30 µm for (M). (N) Depth color‐coded 3D rendering of TH immunostaining in the kidney. Scale bar, 1000 µm. (O) Sectional view of (N), displaying the distribution of TH+ sympathetic fibers in both renal cortex and medulla. Scale bar, 1000 µm. (P) 3D view of a selected region from (N), highlighting dense TH+ fibers within the renal cortex. Scale bar, 400 µm. (Q to R) Enlarged views of the boxed regions in (P), revealing fine details of TH^+^ sympathetic fiber morphology. Scale bars,400 µm for Q (yellow box);100 µm for R (red box).

For vascular mapping, Lycopersicon esculentum (tomato) lectin was injected intravenously into C57 mice before clearing. SynAtlas treatment enabled high‐resolution imaging of whole‐brain and kidney vasculature. In the brain, light‐sheet imaging revealed intact 3D vascular networks and fine capillaries in the olfactory bulb (OB), hypothalamus, and brainstem (Figure 3D,E and movie S5). In the kidney, depth‐coded projections and higher‐magnification views showed the 3D organization of vessels and glomeruli, including fine capillary networks (Figure 3F–H and movie S6).

### 2.5 3D Mapping of Peripheral Sympathetic Nerves with Immunostaining

Peripheral organs are richly innervated by sympathetic nerves, which play essential roles in regulating cardiovascular, metabolic, immune, and endocrine homeostasis. Tyrosine hydroxylase (TH), the rate‐limiting enzyme in catecholamine synthesis, serves as a canonical marker for sympathetic fibers. Aberrant sympathetic innervation has been implicated in hypertension, metabolic syndrome, autoimmune disorders, and cancer, underscoring the importance of 3D mapping of sympathetic networks.

To assess the compatibility of SynAtlas with immunostaining, tissues were first pretreated to enhance antibody penetration. Hemoglobin‐free tissues underwent SynAtlas‐Delipidation, while hemoglobin‐rich tissues were processed using SynAtlas‐Decolorization. Following pretreatment, samples were incubated with a TH‐specific primary antibody for 72 h, followed by a 72 h incubation with a fluorescently labeled secondary antibody. The labeled tissues were subsequently cleared using the two‐step SynAtlas protocol. In small intestine tissue, TH‐positive fibers were localized along the muscle layers, consistent with their known role in modulating intestinal peristalsis (Figure 3I,J). In the ovary, dense sympathetic networks were detected surrounding the medulla and corpus luteum (Figure 3K–M), in agreement with previous findings on sympathetic modulation of ovarian physiology. For kidney samples, depth color coding was applied to the 3D‐rendered TH‐positive signals to better visualize the overall 3D architecture of sympathetic innervation (Figure 3N and movie S7). This representation clearly delineated the spatial distribution of TH‐positive fibers across different renal regions (Figure S9A,B). Optical sectioning further revealed that TH‐positive fibers closely followed renal vasculature, with relatively sparse innervation in the medulla but much denser networks in the cortex (Figure 3O), which was highly consistent with the signal distribution observed via post‐clearing 2D sectioning and immunostaining (Figure S9A,B). Localized magnified views provided higher‐resolution detail, revealing fine perivascular sympathetic fibers intricately distributed along cortical vessels (Figure 3P–R).

Furthermore, to evaluate the penetration and clearing performance of SynAtlas in tissues with a dense extracellular matrix, we applied the pipeline to intact mouse hearts. Combined with the SynAtlas‐Decolorization protocol, whole‐mount TH immunostaining enabled 3D visualization of sympathetic nerve fibers across the epicardial surface and within the deep myocardium (Figure S9C–E). We next examined whether spatial RNA information was preserved after SynAtlas processing. FISH for myosin light chain 2 (*Myl2*) and atrial natriuretic peptide (*Nppa*) revealed their expected ventricular‐ and atrial‐enriched expression patterns [29, 30], respectively (Figure S9F–H). Together, these results demonstrate that SynAtlas enables deep 3D protein imaging while preserving spatial RNA information in intact cardiac tissue, supporting its applicability to organs with a relatively dense extracellular matrix.

### 2.6 Whole‐Organ Multiplexed FISH for 3D Gene Expression Mapping

To demonstrate the capability of SynAtlas for volumetric spatial analysis of gene expression, we combined it with multiplexed fluorescence in situ hybridization (FISH) in the pituitary gland. Leveraging the superior RNA preservation provided by SynAtlas (as evidenced in Figure S3), we implemented a two‐round hybridization chain reaction (HCR) workflow to map a targeted panel of transcripts encoding six major anterior pituitary hormones: Thyroid‐Stimulating Hormone (*Tshb*), Prolactin (*Prl*), Follicle‐Stimulating Hormone (*Fshb*), Adrenocorticotropic Hormone (*Pomc*), Growth Hormone (*Gh*), and Luteinizing Hormone (*Lhb*).

Following SynAtlas clearing and volumetric imaging, whole‐tissue registration based on DAPI produced high‐precision alignment between rounds (Figure S10A–G), demonstrating the exceptional dimensional stability and minimal structural deformation of the tissues during the clearing process. This fidelity enabled the single‐cell reconstruction of all six hormone‐secreting populations within intact pituitaries (Figure 4A–C and movie S8). Magnified views confirmed robust signals across rounds, indicating that the gentle clearing chemistry preserves mRNA targets through stripping and re‐hybridization cycles (Figure 4D–F).

**FIGURE 4:**
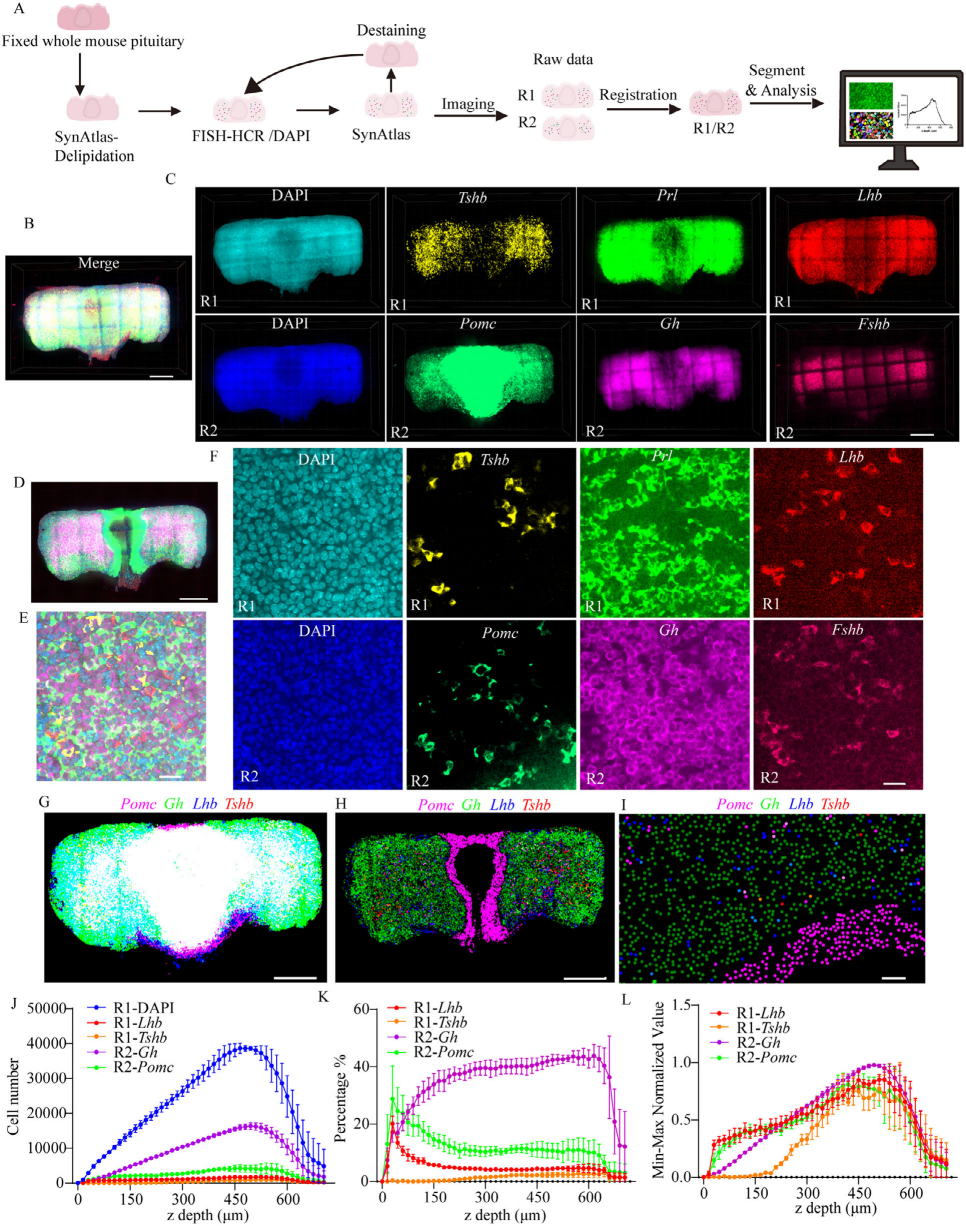
Multiplexed FISH and 3D digital mapping of pituitary hormone‐producing cells using SynAtlas. (A) Schematic workflow illustrating multi‐round multiplexed FISH on whole mouse pituitary glands, followed by image registration and computational data analysis. (B) 3D rendering of the pituitary after two rounds of multiplexed FISH and subsequent registration, showing the overall spatial distribution of hormone‐expressing cells. Scale bar, 500 µm. (C) 3D reconstructions of individual hormone‐producing cell populations from different rounds of labeling, co‐visualized with DAPI‐stained nuclei. Scale bar, 500 µm. (D) Sectional view of the pituitary shown in (B), providing internal visualization of spatially resolved hormone‐expressing cells. Scale bar,500 µm. (E) Enlarged view of a selected region from (D), showing detailed organization of multiple hormone‐producing cell types together with nuclear staining. Scale bar, 30 µm. (F) Channel‐separated images corresponding to (E), displaying distinct fluorescence signals for each hormone marker. Scale bar, 30 µm. (G) 3D digital cell atlas of pituitary hormone cells generated through automated cell segmentation based on multiplexed FISH data. Scale bar, 500 µm. (H) Sectional view of the digital cell atlas in (G), highlighting spatial relationships among segmented cell populations. Scale bar, 500 µm. (I) Enlarged view of a selected region in (H), illustrating the spatial distribution and intermixing patterns of different hormone‐secreting cell types. Scale bar, 50 µm. (J) Quantification of the absolute number of distinct hormone cell types along the dorsoventral axis of the pituitary (*n* = 3 for each group). (K) Proportional distribution of hormone cell types along the dorsoventral axis (*n* = 3 for each group). (L) Min–Max normalized enrichment profiles depicting relative enrichment of hormone cell types along the dorsoventral axis (*n* = 3 for each group).

To move beyond visualization to quantitative spatial analysis, we employed deep learning–based segmentation to identify *Pomc*‐, *Gh*‐, *Lhb*‐, and *Tshb*‐positive cells, constructing a comprehensive 3D digital cell atlas (Figure 4G‐I and Figure S10H–K). Quantitative analysis along the dorsal–ventral (D–V) axis revealed heterogeneous distributions of hormone‐secreting cells. Absolute counts (Figure 4J) showed that total cell numbers increased from the dorsal surface, peaked near the mid‐axis, and declined ventrally. To correct for density differences, we calculated the proportion of each hormone‐positive population relative to total cells (hormone/DAPI ratio) in each D–V section (Figure 4K). This revealed hormone‐specific patterns: *Gh*‐positive cells gradually increased toward ventral regions; *Lhb*‐ and *Pomc*‐positive cells accumulated ventrally before decreasing; and *Tshb*‐positive cells displayed a rise–then–fall trend with confinement near the mid‐axis. Finally, normalization of each hormone cell type to its own maximum abundance (Figure 4L) highlighted their relative enrichment zones: *Tshb*‐positive cells were sharply restricted to a narrow mid‐axis region, *Gh*‐positive cells shifted ventrally, while *Lhb*‐ and *Pomc*‐positive cells were more enriched in dorsal‐to‐mid regions. Notably, all four populations reached their relative density peaks near the central D–V plane, suggesting a conserved mid‐axis functional zoning within the pituitary. These results demonstrate that SynAtlas enables whole‐organ, single‐cell‐resolution multiplexed RNA FISH, providing a scalable framework for targeted spatial gene expression mapping in complex tissues.

### 2.7 Multi‐scale 3D Atlas Construction in Whole Mice and Large Blocks

To assess whole‐body applications, we established a three‐step workflow—decalcification, decolorization and RI matching—for SynAtlas‐based clearing of bone‐containing specimens. In 3‐week‐old Thy1‐EYFP mice, the entire protocol was completed within 72 h (Figure 5A). Light‐sheet imaging revealed well‐preserved peripheral YFP‐labeled nerves, including facial and musculocutaneous branches (Figure 5B,C).

**FIGURE 5:**
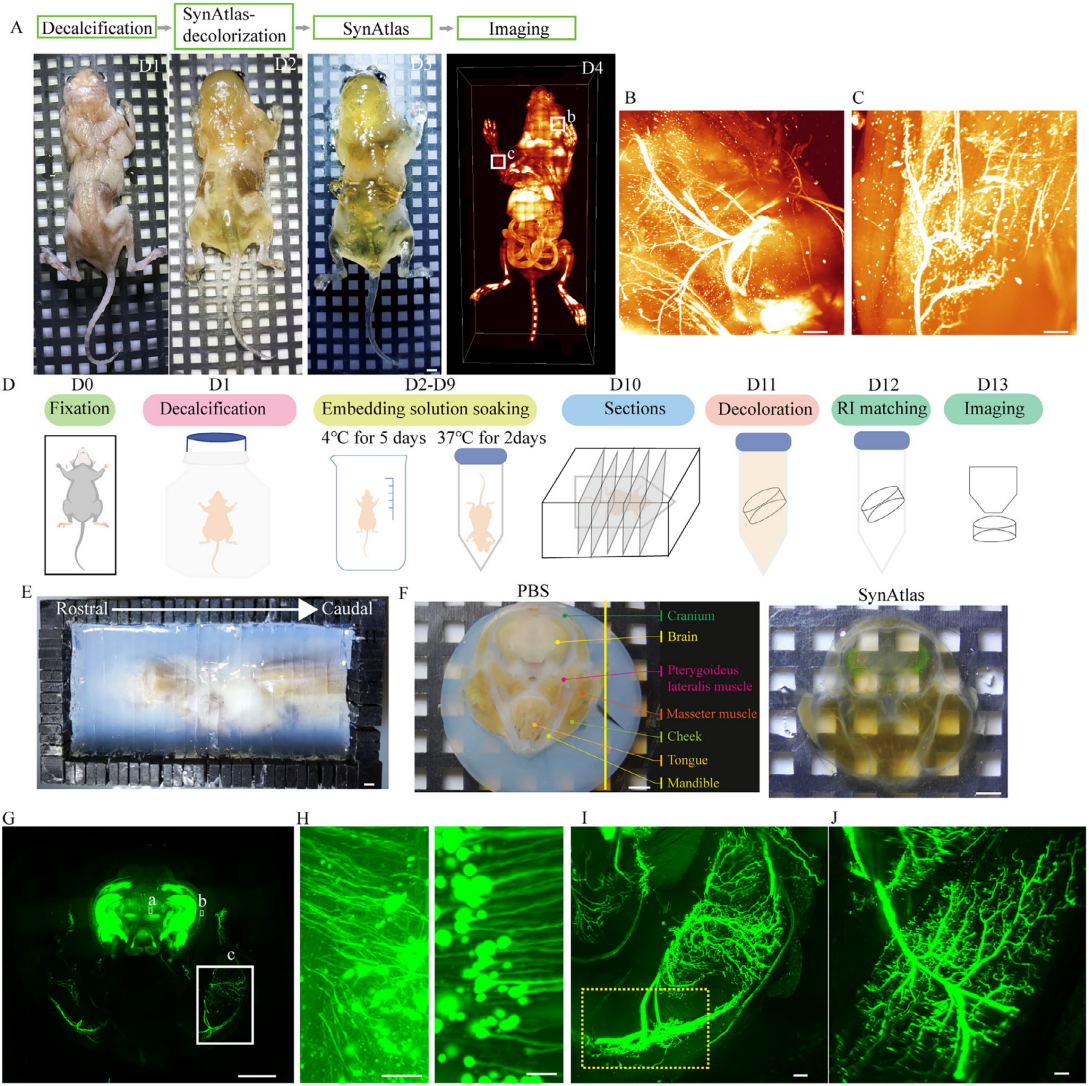
Whole‐body and large‐scale tissue clearing using SynAtlas. (A) Schematic of the three‐step whole‐body clearing and imaging workflow applied to juvenile Thy1‐EYFP mice. Scale bar, 2.5 mm. (B to C) Enlarged views of the boxed regions in (A), corresponding to the lowercase labels b and c, showing well‐preserved YFP‐labeled facial nerves (B) and musculocutaneous nerves (C) following SynAtlas clearing. Scale bars, 700 µm. (D) Workflow illustrating embedding, sectioning, and SynAtlas‐based clearing of adult mouse tissue slabs. (E) Representative image of an embedded adult Thy1‐EYFP mouse after sectioning into tissue slabs prior to clearing. Scale bar, 2 mm. (F) Comparison of a 2‐mm‐thick tissue slab before and after SynAtlas clearing, demonstrating the significant increase in optical transparency. Scale bar, 2.5 mm. (G) 3D light‐sheet rendering of a cleared 2‐mm tissue slab shown in (F), revealing detailed neural architectures across large tissue volumes. Scale bar, 3000 µm. (H) Enlarged views of regions a and b in (G), showing fine neural fibers in the brainstem (a) and somata in the cerebral cortex (b). Scale bars, 200 µm (left panel); 50 µm (right panel). (I) Enlarged view of region c in (G), displaying a 3D rendering of facial nerve bundles with high structural fidelity. Scale bar, 400 µm. (J) Further magnified view of the boxed region in (I), highlighting detailed structures of facial nerve fibers. Scale bar, 150 µm.

For adult mice, full‐body imaging was limited by chamber size, so specimens were embedded and sectioned into 2‐mm slabs for clearing and imaging (Figure 5D,E). SynAtlas maintained high transparency in these large blocks (Figure 5F). Light‐sheet imaging revealed extensive YFP‐labeled networks throughout, with higher‐magnification views resolving nerve bundles and neuronal somata in the brain and peripheral nerves in facial regions (Figure 5G–J and movie S9).

These data show that SynAtlas supports multi‐scale 3D imaging from thick organ slices to whole‐body neural networks, enabling systematic anatomical reconstruction of circuits across scales.

### 2.8 3D Cellular Mapping and Multi‐Round Phenotyping in Human Brain Tissue

Finally, we evaluated the utility of SynAtlas for human neuroscience. Archival human brain tissues present significant challenges for optical clearing due to prolonged formalin fixation and the high scattering nature of dense myelination. SynAtlas proved highly effective for these challenging specimens. Human cortical sections (100–1000 µm) were rendered transparent within minutes to hours depending on thickness, with 1‐mm blocks achieving complete clarity within 24 h (Figure 6A).

**FIGURE 6:**
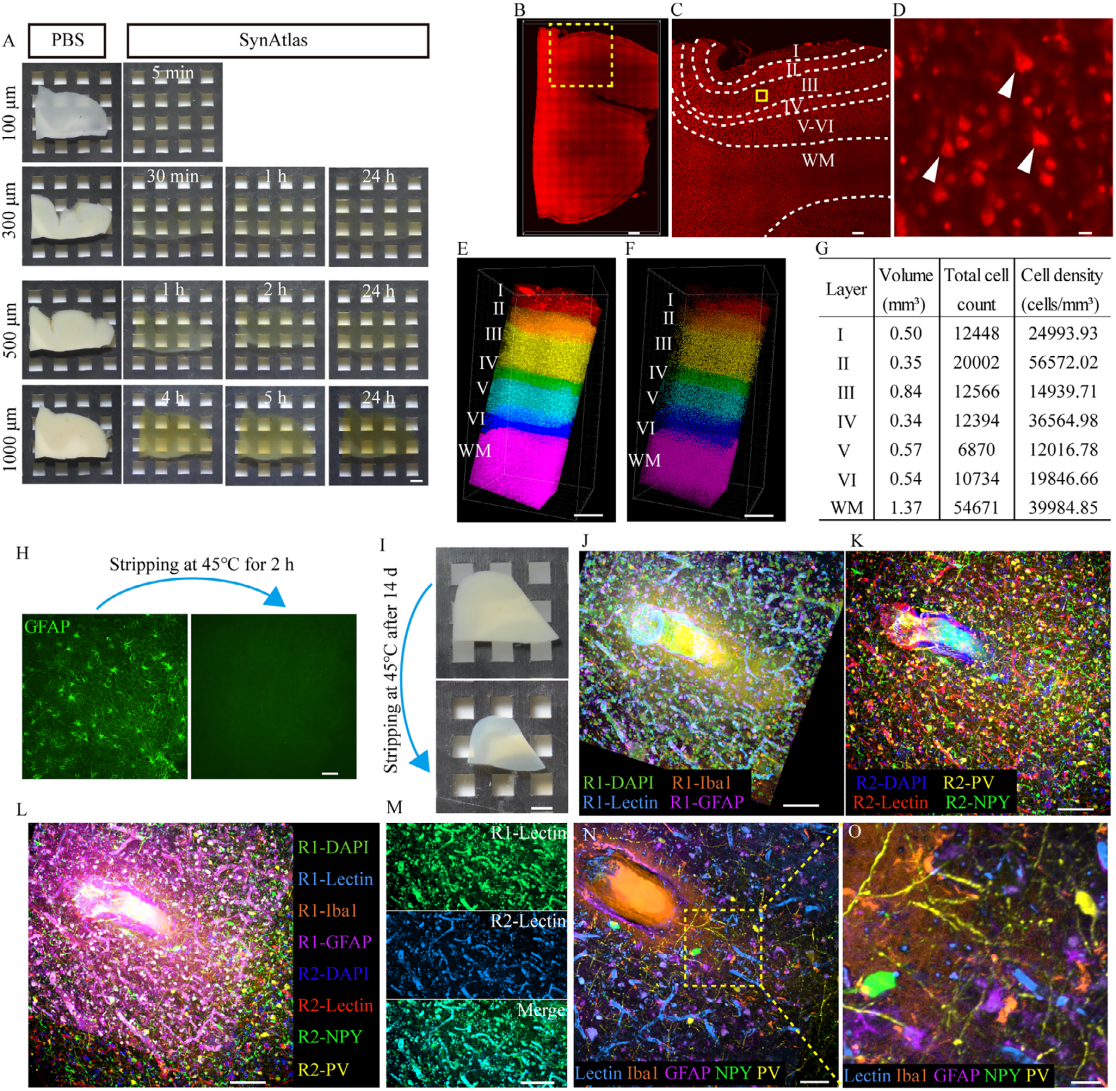
Application of SynAtlas to human brain tissue for 3D cellular mapping. (A) Representative clearing results of human cortical sections with different thicknesses after various durations of SynAtlas treatment. Scale bar, 2.5 mm. (B) 3D rendering of a 1‐mm‐thick PI‐stained human temporal lobe block imaged with light‐sheet microscopy. Scale bar, 1000 µm. (C) Enlarged view of the boxed region in (B), showing well‐preserved cortical lamination patterns in the temporal lobe. Scale bar, 200 µm. (D) Higher‐magnification view of the boxed region in (C), revealing PI‐labeled pyramidal neurons within cortical layer III, as indicated by white arrows. Scale bar, 15 µm. (E) 3D reconstruction of a selected volume from (B), with distinct cortical layers manually annotated using different colors. Scale bar, 500 µm. (F) Automated detection of cells in (E) using Imaris, with different cortical layers labeled in distinct colors. Scale bar, 500 µm. (G) Quantification of total cell numbers across different cortical layers based on Imaris segmentation results from (F). (H) Immunofluorescence staining of GFAP in human brain tissue following SynAtlas‐Decolorization treatment, showing effective labeling and subsequent antibody stripping. Scale bar, 50 µm. (I) Stereomicroscope images of a 300‐µm‐thick human brain section before and after 14 days of treatment with stripping buffer. Scale bar, 2.5 mm. (J) First‐round immunostaining of SynAtlas‐Decolorization–treated 100‐µm human brain tissue with Iba1, GFAP, DAPI, and Lectin‐594. Scale bar, 100 µm. (K) Second‐round immunostaining of the same sample after antibody stripping, showing PV, NPY, DAPI, and Lectin‐594 labeling. Scale bar, 100 µm. (L) Registration of the first‐round (J) and second‐round (K) immunostaining datasets using the Lectin‐594 vascular signal as a reference. Scale bar, 100 µm. (M) Merged view showing overlapping Lectin‐594 signals from both rounds after registration. Scale bar, 100 µm. (N) Spatial co‐registration of Iba1, GFAP, NPY, PV, and Lectin‐594 signals from both staining rounds. Scale bar, 50 µm. (O) Magnified view of the yellow boxed region in (N), showing detailed local distributions of the labeled cell types and vessels. Scale bar, 15 µm.

In 1‐mm temporal lobe blocks, PI labeling followed by SynAtlas clearing enabled uniform visualization of cytoarchitecture across cortical layers, revealing distinct anatomical boundaries (Figure 6B,C). High‐resolution volumetric imaging resolved fine neuronal morphologies, including the pyramidal cells in layer III (Figure 6D). Depth‐coded renderings highlighted the 3D organization of cortical layers and the underlying white matter (Figure 6E), allowing for the generation of digital 3D cellular distribution maps via automated segmentation (Figure 6F,G).

Crucially, SynAtlas offers a streamlined alternative to hydrogel‐based methods for multi‐round labeling. While crosslinking‐based techniques (e.g., SHIELD) provide robust stability, they often necessitate laborious hydrogel embedding protocols and harsh stripping conditions. In contrast, the hydrogel‐free nature of SynAtlas allows for rapid antibody diffusion and efficient stripping using mild buffers. To validate the efficiency of antibody removal, we immunostained human cortical tissue for Glial Fibrillary Acidic Protein (GFAP). Following a 2 h incubation in a mild stripping buffer, the fluorescence signal was completely eliminated (Figure 6H). To determine whether the stripping procedure affected subsequent antigen recognition, we performed an independent antigen‐preservation experiment using serial human brain sections. Compared with non‐stripped control sections, sections pretreated with the stripping buffer showed no reduction in subsequent GFAP immunostaining; instead, GFAP fluorescence intensity was significantly increased (Figure S11A–C). These results indicate that the loss of fluorescence immediately after stripping reflects antibody removal rather than loss of GFAP antigenicity. The enhanced GFAP staining following stripping may result from guanidine hydrochloride and β‐mercaptoethanol in the stripping buffer further disrupting the densely fixed tissue structure, thereby increasing epitope accessibility.

To ensure that repeated stripping does not compromise tissue integrity, we subjected the samples to a rigorous stability test: long‐term incubation (14 days) in the same mild stripping buffer. This duration, equivalent to approximately 168 stripping cycles, did not alter gross morphology (Figure 6I), confirming that SynAtlas‐processed tissue retains robust structural integrity without the need for additional covalent hydrogel embedding.

Leveraging this stability, we performed multi‐round immunostaining on 100‐µm human cortical sections. The gentle stripping buffer effectively removed antibodies from the first round (Iba1 + GFAP) without compromising antigenicity for the second round (Neuropeptide Y [NPY] + Parvalbumin [PV]) (Figure 6J,K). Registration using vascular landmarks (Lectin‐594) achieved precise alignment (Figure 6L,M), enabling the spatial integration of glial and neuronal subtypes within the same tissue volume (Figure 6N,O). These results confirm that SynAtlas is a versatile platform for multi‐modal 3D reconstruction of human brain organization, applicable to valuable archival clinical samples.

## 3 Discussion

In this study, we developed SynAtlas, a safe and versatile tissue clearing platform guided by an “orthogonal synergy” design principle that simultaneously addresses the three primary orthogonal optical barriers (lipid scattering, protein hydration heterogeneity, and bulk RI mismatch) to tissue transparency using the synergy effect of functionally distinct chemical modules. SynAtlas achieves rapid, high‐quality tissue clearing while maintaining excellent biocompatibility and broad applicability. Our results demonstrate that this orthogonal approach enables clearing of thin sections within minutes and whole organs within 2 days, preserves diverse molecular labels including fluorescent proteins, nucleic acids, and immunostaining epitopes, and supports advanced applications such as whole‐organ multiplexed RNA FISH and multi‐round immunophenotyping of archival human brain tissue.

The three primary optical barriers to tissue transparency—lipid‐induced scattering, protein hydration heterogeneity, and bulk refractive index (RI) mismatch—arise from distinct physicochemical mechanisms and spatial scales, making them orthogonal [24, 31, 32, 33]. Lipid‐induced scattering primarily stems from Mie scattering at lipid–aqueous interfaces (e.g., myelin sheaths), driven by large RI contrasts. Protein hydration heterogeneity produces sub‐wavelength RI fluctuations from uneven hydration of protein domains, causing Rayleigh‐like diffuse scattering even after lipid removal. Bulk RI mismatch occurs at the macroscopic level due to the difference between average tissue RI and the imaging medium, amplifying internal multiple scattering and limiting light penetration. These independent barriers mean that addressing only one or two is insufficient for deep, high‐fidelity transparency. Guided by this understanding of orthogonal optical barriers, we developed SynAtlas using the principle of orthogonal synergy: integrating three chemically distinct modules—a hydrotropic amphiphile for lipid solubilization, a hydrogen‐bond modulator for uniform protein hydration, and a high‐RI agent for bulk index matching—that are designed to address distinct optical barriers. This rational design achieves rapid clearing with excellent preservation of tissue integrity and molecular signals. Crucially, this chemical consistency enables parallel handling of multiple optical barriers, establishes a streamlined kinetic efficiency that simplifies experimental procedures and increases kinetic efficiency, and distinguishes SynAtlas from the disjointed multi‐step protocols of widely adopted serial processing. Unlike traditional methods that require extensive washing between incompatible steps [14, 15, 18], SynAtlas utilizes chemically compatible reagents. For whole organs, the delipidation step increases tissue permeability, allowing the high‐RI solution to penetrate rapidly without intermediate transitions. This direct workflow significantly accelerates clearing and minimizes tissue damage caused by solvent changes.

In contrast to empirical optimization, the orthogonal synergy principle offers a conceptual scaffold for the rational design of tissue clearing media. While existing methods such as EZ Clear or OptiMuS utilize chemically analogous reagents (e.g., urea and iohexol) [18, 19], they are typically presented as fixed compositions derived from specific screening conditions. This formulation dependence limits their adaptability to novel experimental constraints. Conversely, SynAtlas functions as a modular physicochemical framework. As demonstrated by the rational substitution of core components—such as swapping urea for NMDG, replacing iohexol with alternative high‐RI agents like antipyrine or uridine, or even adopting high‐RI mixtures (e.g., antipyrine/nicotinamide) and ionic liquids (e.g., NAPID/PA)—or the seamless integration of decolorizing reagents such as N‐butyldiethanolamine to address the distinct optical barrier of heme‐induced absorption—the orthogonal synergy approach enables the deductive engineering of clearing variants tailored to specific requirements (e.g., cost‐efficiency, dye compatibility, cryopreservation capability, or biosafety). This establishes a generalizable logic for formulation development, reducing reliance on random combinatorial screening. In this study, our systematic screen of 1600 reagents was explicitly driven by safety constraints to identify low‐hazard candidates within the targeted chemical classes. This ensures that the final orthogonal triad not only achieves optical synergy but also strictly adheres to the biosafety standards required for routine open‐bench applications.

A defining characteristic of the SynAtlas system is its exceptional versatility and broad compatibility, which distinguishes it from existing clearing modalities. This platform is effective across a vast range of physical scales—from thin tissue sections to whole organs and even intact bodies—and is applicable to diverse biological tissues derived from mice and humans. Beyond structural clearing, SynAtlas demonstrates robust compatibility with a wide array of molecular probes, including endogenous fluorescent proteins, small‐molecule dyes, and immunofluorescent labels. Crucially, it enables multiplexed fluorescence in situ hybridization (FISH) and multi‐round immunofluorescence without the need for complex hydrogel embedding, thereby facilitating the construction and quantitative analysis of high‐resolution 3D atlases at the whole‐organ level. Furthermore, this comprehensive performance is achieved using low‐hazard reagents through a simple, efficient workflow that preserves tissue structural integrity even under cryogenic storage conditions. While specific existing methods have optimized individual aspects of this spectrum—such as speed, transparency, or molecular retention—there remains a lack of a single methodology that simultaneously delivers such widespread compatibility and applicability. SynAtlas effectively bridges this gap, offering a unified solution for multi‐modal volumetric imaging.

A distinctive and unexpected physicochemical property of SynAtlas is its behavior at cryogenic temperatures. Unlike standard aqueous buffers that crystallize into opaque ice, SynAtlas undergoes a transition into a transparent, glass‐like solid upon rapid cooling—a phenomenon akin to vitrification. This behavior parallels the cryo‐preservation capabilities recently reported in the VIVIT method [23]; however, SynAtlas achieves this state through a distinct chemical mechanism. While VIVIT relies on ionic liquids to maintain a glassy state, SynAtlas leverages the synergistic high concentrations of urea and iohexol. These components likely form an extensive hydrogen‐bond network that disrupts the lattice formation of water molecules, thereby effectively suppressing ice crystallization [34, 35, 36]. Our freeze‐thaw analysis confirmed that this mechanism protects tissue ultrastructure, as pore architectures in SynAtlas‐treated tissues remained indistinguishable from controls. In routine workflows, storing cleared samples at room temperature or 4°C is generally sufficient to maintain the stability of endogenous fluorescence and protein epitopes. However, for experiments requiring subsequent nucleic acid detection (such as in situ hybridization), the inherent chemical susceptibility of RNA to degradation limits long‐term storage at conventional temperatures, and its stable preservation typically requires low‐temperature environments of −20°C or −80°C. The cryogenic properties of SynAtlas accommodate this need, offering the possibility of establishing biobanking for cleared tissues. In this workflow, cleared tissues can be directly frozen and archived after imaging, then thawed for re‐imaging, tissue sectioning, or detection of fragile markers (particularly RNA), while maintaining morphological and molecular integrity. Furthermore, integrating the ionic liquid components utilized in VIVIT into our orthogonal synergy framework achieved rapid mouse half‐brain clearing within 24 h, while successfully maintaining sample transparency at cryogenic temperatures. This drastically reduces processing times compared to the multi‐day schedule required by the standard VIVIT protocol, thereby significantly enhancing experimental throughput. Notably, incorporating the antipyrine/nicotinamide mixture as the high‐RI matching module within the orthogonal synergy framework also conferred a similar cryo‐stable transparency to the cleared tissues. Given that an antipyrine/nicotinamide blend is unlikely to form an ionic liquid, it presumably operates as a deep eutectic solvent (DES) that suppresses crystallization at low temperatures. Collectively, these findings significantly expand the applicability of the orthogonal synergy framework. They demonstrate that targeted optical and physicochemical barriers in tissue clearing can be circumvented not only by optimizing specific single molecules but also by employing functional mixtures, offering unprecedented flexibility for the rational design of next‐generation clearing media.

Constructing high‐resolution, single‐cell atlases at the whole‐organ scale is fundamental to decoding systemic physiological and pathological states. Our study demonstrates that SynAtlas facilitates this by integrating transgenic, viral, and dye‐based labeling to generate comprehensive whole‐brain cell distribution and projection maps. By enabling precise registration to the ABA, SynAtlas provides a streamlined and rapid alternative to complex pipelines like CUBIC‐Atlas or SOLID for quantitative volumetric analysis [22, 37]. Beyond the central nervous system, we extended this capability to peripheral organs, constructing 3D sympathetic innervation maps via immunofluorescence to facilitate neuro‐organ interaction studies. Furthermore, we achieved a significant methodological advance by performing multi‐round, multiplexed FISH on intact pituitary glands. This enabled us to construct—for the first time, to our knowledge—a spatial atlas of six hormone‐secreting cell types, revealing their distinct heterogeneous distributions along the dorsal–ventral axis. While techniques such as EASI‐FISH or cycleHCR support multi‐round imaging, they typically rely on laborious hydrogel embedding and specific RNA‐anchoring reagents [38, 39]. In contrast, SynAtlas enables robust multiplexing in standard PFA‐fixed tissues, drastically reducing experimental complexity and enhancing scalability. Notably, despite the pituitary gland's significantly higher cell density compared to the brain, we observed near‐perfect nuclear registration across hybridization rounds. This confirms that SynAtlas maintains exceptional dimensional stability and minimal distortion even after repeated clearing and staining cycles, ensuring the accuracy required for single‐cell analysis in dense tissue environments.

For human neuroscience, SynAtlas excels with archival formalin‐fixed tissues, which are notoriously difficult to clear due to prolonged fixation and dense myelination [25, 26]. Dense cortical blocks (up to 1 mm) clear within 24 h, enabling uniform nucleic acid labeling and high‐resolution cytoarchitectural imaging. Crucially, the hydrogel‐free nature allows efficient, mild antibody stripping (complete removal in 2 h with no structural compromise even after simulated 168 cycles), supporting multi‐round immunostaining (e.g., Iba1/GFAP followed by NPY/PV) with precise vascular‐landmark registration. This capability offers substantial value for precious clinical samples, enabling integrated 3D phenotyping of glial and neuronal subtypes without the complexity or distortion risks associated with crosslinking‐based methods. In recent years, multi‐round immunostaining techniques have made significant advances. Methods such as CLARITY, SWITCH, SHIELD, and MAP enhance tissue tolerance to harsh destaining conditions through hydrogel embedding, thereby enabling multiple rounds of immunostaining [15, 17, 40, 41]. In contrast, our approach employs mild destaining conditions that allow multi‐round labeling even on standard PFA‐fixed tissues. This simplifies the workflow, seamlessly integrates multi‐round immunostaining with the SynAtlas method, and thereby improves its convenience and broad applicability.

Despite its robust performance, certain limitations warrant consideration. First, the inclusion of iohexol entails a higher cost compared to low‐cost reagents like sucrose. However, for high‐value samples such as transgenic whole organs or human biopsies, the gains in speed, signal preservation, and cryo‐stability likely outweigh the reagent expenses. Moreover, our demonstration that substituting iohexol with antipyrine or other high‐RI mixtures can achieve comparable transparency suggests that further exploration of alternative low‐cost, high‐RI reagents may reduce expenses for large‐scale applications. Second, while SynAtlas significantly accelerates the optical clearing process, the kinetics of antibody penetration in whole organs remains governed by passive diffusion. Although compatible with deep‐tissue labeling, immunostaining large‐volume specimens still requires extended incubation times—a physical constraint shared by all immersion‐based approaches. Future integration with active transport methods, such as electrophoresis, could help resolve this staining bottleneck. Finally, the current protocol employs a high‐osmolarity RI‐matching solution. While we observed minimal distortion in adult tissues, extremely fragile specimens (e.g., early‐stage embryos) might experience transient osmotic stress. For such delicate samples, introducing a graded concentration series during the RI‐matching step may be necessary to ensure optimal morphological preservation. Furthermore, although the current SynAtlas formulation uses functionally distinct reagents to target different sources of light scattering and absorption, individual components may simultaneously modulate multiple optical barriers. This multifunctional capacity opens the possibility of designing even simpler and more efficient clearing reagents in the future. A broader comparison of SynAtlas with representative tissue‐clearing methods, including their workflows, key reagents, clearing time, tissue deformation, molecular preservation, and application compatibility, is summarized in Table S5.

In summary, SynAtlas demonstrates that rationally targeting orthogonal optical barriers through synergistic, functionally distinct chemical modules can produce a powerful, unified clearing platform that balances speed, safety, molecular fidelity, dimensional stability, and multi‐modal compatibility. By moving beyond empirical optimization toward a conceptual design principle, this work not only provides a practical tool for high‐throughput volumetric imaging but also establishes a generalizable framework for the rational engineering of next‐generation biocompatible clearing chemistries. We anticipate that the orthogonal synergy approach will facilitate the development of tailored clearing agents for emerging applications, including large‐scale spatial transcriptomics in human archival tissues and high‐resolution 3D pathology in clinical settings. Ultimately, SynAtlas offers a pathway to make deep, multi‐scale 3D biological mapping more accessible, reproducible, and impactful across neuroscience and translational research.

## 4 Experimental Section

### 4.1 Animals

The experimental animals and experimental protocols used in this study were approved by the Animal Care and Use Committee of Anhui Medical University (LLSC20241768). The mouse strains used in this study ranged in age from 3 weeks to 12 months, including C57BL/6J, Thy1‐EYFP, CRH‐Cre;Ai9, VIP‐Cre;Ai6, and ChAT‐Cre;Ai6. The mice were housed in a stable environment with controlled temperature (22°C–23°C) and humidity (60%), following a circadian rhythm cycle (12/12 h light/dark), and had unrestricted access to food and water.

### 4.2 Sample Preparation

Mice were anesthetized with 0.8% sodium pentobarbital and transcardially perfused with 15 mL of 0.1 M phosphate buffer solution (PBS), followed by 20 mL of 4% paraformaldehyde (PFA, dissolved in 0.1 M PBS) for fixation. The brain, lungs, liver, spleen, heart, intestines, and kidneys were carefully dissected and post‐fixed in 4% PFA at 4°C for 24 h. Tissue sections ranging from 100 µm to 2 mm in thickness were obtained using a vibratome (Leica VT1200S) and stored at 4°C in PBS containing 0.02% sodium azide.

Human brain tissues were obtained from the China National Health and Disease Human Brain Tissue Resource Center (Hangzhou, China). All materials were collected from donors who had provided written informed consent for a brain autopsy and permitted their clinical information for research purposes. Formalin‐fixed tissue samples from the inferior temporal gyrus were washed three times with PBS (1 h each) and sectioned into 100–2000 µm slices using a Leica VT1200S vibratome. Sections were stored at 4°C in PBS containing 0.02% sodium azide.

### 4.3 Screening and rational design of SynAtlas reagents

Potential chemical components were screened from a comprehensive library of 1631 CUBIC chemical entries previously reported by Tainaka and colleagues [14]. Physicochemical and economic parameters—including the lipid solubility score (LSS), decoloring score (DS), refractive index (RI), and cost per unit—were initially compiled into a standardized database (Table S1, “Database” sheet). To ensure economic feasibility for high‐throughput and large‐scale volumetric imaging, reagents with a unit price exceeding 600 JPY were eliminated, yielding 1150 candidates (Table S1, “Cost600” sheet). Subsequent data curation to remove duplicate entries and those with incomplete chemical profiles narrowed the library to 1093 valid candidates (Table S1, “Filtered1093” sheet).

To construct a strictly low‐hazard formulation compatible with routine laboratory environments, a two‐tier biosafety filtering pipeline was implemented using GHS classifications and acute toxicity data retrieved from PubChem, Sigma‐Aldrich Safety Data Sheets (SDS), and TCI Chemicals SDS databases. First, all reagents bearing the GHS signal word “Danger” were excluded, retaining 700 low‐hazard candidates categorized as “Warning” or “Not a hazardous substance or mixture” (Table S1, “LowHazard700” sheet). Second, acute oral toxicity was evaluated: candidates bearing a “Warning” designation were excluded if their oral LD_50_ in rats was below 2000 mg/kg or unrecorded. This rigorous safety screening established a foundational pool of 293 low‐hazard candidate reagents (Table S1, “LowToxic293” sheet; Figure S1A).

Guided by the orthogonal synergy framework, candidates from this 293‐reagent pool were systematically allocated to three functional modules targeting independent optical barriers:

1. Refractive index (RI) matching module: Candidates were ranked by RI. High‐concentration inorganic salts were intentionally excluded to prevent severe osmotic stress, tissue shrinkage, and protein salting‐out effects. From the top‐performing organic candidates, nicotinamide, creatinine, 3,6‐dithia‐1,8‐octanediol, uridine, 1‐(2‐hydroxyethyl)piperazine, 1,3‐dimethyl‐5‐pyrazolone, and iohexol (Histodenz) were selected for experimental validation (Table S1, “Top_RI” sheet).
2. Lipid removal (amphiphilic) module: Candidates were ranked by their LSS scores. From the top performers, cost‐effective amphiphilic agents with distinct molecular mechanisms—including Tween 80, sodium dodecylbenzenesulfonate (SDBS), Triton X‐100, and 1,2‐hexanediol—were selected (Table S1, “Top_LSS” sheet).
3. Hydrogen‐bond modulation module: As the CUBIC library lacked a dedicated quantitative parameter for protein hydration, urea and established protein‐denaturing analogs (guanidine hydrochloride, formamide, thiourea, and m‐xylylenediamine [MXDA]) were evaluated based on literature. Crucially, only urea satisfied our prior safety criteria (lacking a “Danger” signal word and exhibiting an oral LD_50_ ≥ 2000 mg/kg), whereas alternative analogs were eliminated due to high toxicity or severe hazard classifications.

Finally, candidate reagents selected from the three functional modules were experimentally evaluated through stepwise formulation optimization. Chemical compatibility, particularly solubility, was first assessed to exclude physically incompatible formulations, whereas tissue‐clearing performance served as the primary criterion for formulation selection. Representative compatible formulations were further quantitatively compared by spectral transmittance measurements across 400–800 nm.

In this study, “biosafe” is defined based on two specific safety criteria: (i) the absence of a GHS “Danger” signal word classification, and (ii) an acute oral LD_50_ (rat) > 2000 mg/kg. These criteria exclude reagents associated with severe acute toxicity, flammability, or corrosivity that would preclude routine open‐bench use. Importantly, this definition does not constitute a comprehensive toxicological assessment; chronic toxicity, dermal and respiratory irritation, carcinogenicity, environmental impact, and waste‐disposal requirements were not evaluated in this screening. Reagents that pass this initial safety filter are referred to as “biosafe” throughout this work to indicate their suitability for standard laboratory handling.

### 4.4 Preparation of SynAtlas Reagents

SynAtlas comprises three working solutions with the following formulations:

1. SynAtlas‐Delipidation: 10% (v/v) 1,2‐hexanediol and 24% (w/v) urea in distilled water. Store at room temperature.
2. SynAtlas‐Decolorization (Auxiliary Module for Heme‐rich Tissues): 10% (v/v) 1,2‐hexanediol, 24% (w/v) urea, supplemented with 20% (v/v) N‐butyldiethanolamine in distilled water. (N‐butyldiethanolamine carries a GHS “Danger” signal word and should therefore be handled according to the appropriate SDS‐recommended safety precautions. Quadrol or N‐methyldiethanolamine may be used as lower‐hazard alternatives where desired.) Store at room temperature.
3. SynAtlas: 10% (v/v) 1,2‐hexanediol, 24% (w/v) urea, and 60% (w/v) iohexol in distilled water. Store at room temperature.

### 4.5 SynAtlas Clearing Protocols

1. Tissue Sections: Tissue sections (≤ 2 mm) were immersed directly in SynAtlas solution at room temperature or 37°C with gentle agitation until complete transparency was achieved.

2. Intact Organs: Whole organs were first incubated in SynAtlas‐Delipidation for 24 h at 37°C to remove lipids. For heme‐rich organs (e.g., kidney, spleen), SynAtlas‐Decolorization was used to facilitate both delipidation and decolorization. Samples were then transferred to SynAtlas solution and incubated at 37°C for an additional 24 h for RI matching and complete transparency.

3. Whole‐Body Specimens: Whole‐body clearing was performed using one of two approaches, depending on specimen size and imaging requirements:

- Direct whole‐body clearing: Following skin and fur removal, the specimen was decalcified in 20% ethylenediaminetetraacetic acid (EDTA, pH adjusted to 7.4 with NaOH) at 37°C for 24 h. The tissue was then sequentially incubated in SynAtlas‐Decolorization solution for 24 h at 37°C and then with SynAtlas solution at 37°C until complete transparency was achieved.
- Large‐sample embedding and sectioning: Specimens were first decalcified in 20% EDTA (pH 7.0) at 37°C for 24 h, then washed three times in PBS (8 h each) to remove residual EDTA. Samples were embedded in a hydrogel solution containing 20% (w/v) BSA, 4% (w/v) acrylamide, 0.05% (w/v) methacrylamide, and 0.25% (w/v) VA044 initiator at 4°C for 5 days, followed by polymerization at 37°C for 48 h. The embedded specimen was subsequently sectioned into 2‐mm‐thick slices using a custom slicing mold. These slices were first incubated in SynAtlas‐Decolorization solution at 37°C for 24 h to remove residual pigments, followed by immersion in SynAtlas solution at 37°C for complete tissue clearing.

### 4.6 Tissue Clearing with Other Methods

CUBIC: Samples were washed in PBS (RT, 1 h × 3) and immersed in CUBIC‐1 (25 wt.% urea, 25 wt.% Quadrol, 15 wt.% Triton X‐100, distilled water) at 37°C with gentle shaking (80 rpm). CUBIC‐1 was replaced every 2 days, and incubation continued for 7 days for delipidation. Samples were then washed in PBS (12 h) and incubated in CUBIC‐2 (25 wt.% urea, 50 wt.% sucrose, 10 wt.% triethanolamine, distilled water) at 37°C for 2 days with shaking under dark conditions [42]. CUBIC‐L/R: Samples were washed in PBS (RT, 1 h × 3), incubated in CUBIC‐L (10% N‐butyldiethanolamine and 10% Triton X‐100 in distilled water) at 37°C for 7 days with shaking (80 rpm), and the solution was replaced every 2 days. After PBS washing (12 h), samples were transferred to CUBIC‐R (45 wt.% antipyrine, 30 wt.% nicotinamide, distilled water; pH 8–9 adjusted with N‐butyldiethanolamine) and incubated at 37°C for 2 days [14]. EZ Clear: Samples were washed in PBS (RT, 1 h x 3) and incubated in lipid removal solution (50% v/v tetrahydrofuran containing 250 ppm butylated hydroxytoluene in distilled water) at room temperature for 16 h with shaking. After being rinsed with distilled water four times (1 h each at RT), samples were transferred into EZ View solution (80% iohexol, 7 M urea, and 0.05% sodium azide in 0.02 M sodium phosphate buffer, pH 7.4) and incubated at room temperature for 24 h with gentle shaking under dark conditions to achieve transparency [18]. OptiMuS: Samples were washed in PBS (RT, 1 h x 3) and incubated in OptiMuS clearing solution (75% iohexol, 10% D‐sorbitol, and 4 M urea dissolved in 100 mM Tris‐EDTA buffer, pH 7.5) at 37°C with shaking for 3 days to achieve transparency [19]. VIVIT: Samples were washed in PBS (RT, 1 h x 3) and sequentially incubated in DIDC solution at 37°C for 6 days, SOS solution at 10°C for 24 h, and RIL solution at 10°C for 2 h, followed by 4°C until the tissue sank (24 h). Finally, samples were transferred into 70% APIL solution until sinking (24 h) and equilibrated in 83% APIL solution at 37°C for 24 h until completely translucent for imaging. All specialized clearing and refractive index‐matching solutions (DIDC, SOS, RIL, and APIL) were prepared strictly according to the formulations described in the original VIVIT protocol [23].

### 4.7 Fluorescence Preservation Test (YFP Retention)

Brain slices (100 µm thick) from Thy1‐EYFP mice were mounted onto adhesion microscope slides and sealed with either PBS or SynAtlas solution. Imaging was performed immediately after mounting using identical microscope settings for all samples. The fluorescence intensity of YFP signals was quantified using ImageJ.

### 4.8 Nucleic Acid Preservation Test

To evaluate nucleic acid preservation, tissues cleared with SynAtlas, EZ Clear, OptiMuS, CUBIC, CUBIC‐L/R, or VIVIT were initially equilibrated in PBS overnight at 4°C to thoroughly wash out residual clearing reagents. The washed tissues were subsequently sectioned into 100 µm slices using a vibratome. PBS‐treated samples stored at 4°C served as controls. All sections were washed three times in PBST (10 min each) and stained at room temperature for 30 min with DAPI and propidium iodide (PI) (1:1000 dilution) to label DNA and total nucleic acids, respectively. Within somatic cells, the PI signal predominantly reflects the high abundance of cellular RNA. The stained slices were then imaged under identical laser acquisition conditions using a spinning‐disk confocal microscope, and the resulting PI fluorescence intensities were quantified using ImageJ.

For the fluorescence in situ hybridization assays, multiplexed FISH was performed using third‐generation hybridization chain reaction (HCR v3.0) technology. Split‐initiator probe sets targeting *Sst* and *Vglut1* were diluted in hybridization buffer (30% formamide, 5x SSC, 0.1% Tween‐20) and incubated with the tissue sections at 37°C for 24 h. The samples were then washed three times in hybridization buffer (0.5 h per wash, 37°C), followed by a single wash in 5x SSC containing 0.1% Tween‐20 for 0.5 h. Fluorescent hairpins (B1‐Alexa647 and B2‐Alexa488) were subsequently hybridized in 5x SSC containing 0.1% Tween‐20 at room temperature for 24 h in the dark. Post‐hybridization, the samples were washed three times in 5x SSC containing 0.1% Tween‐20 (10 min each, room temperature). Nuclei were counterstained with DAPI (1 ug/mL in PBS) for 30 min in the dark. Finally, tissues were imaged using a spinning‐disk confocal microscope. The specific HCR probes utilized in this study are listed in Table S4.

### 4.9 Evaluation of Antigen Epitope Preservation

To evaluate antigen epitope preservation, tissues cleared with SynAtlas, EZ Clear, OptiMuS, CUBIC, CUBIC‐L/R, or VIVIT were initially equilibrated in PBS overnight at 4°C to thoroughly wash out residual clearing reagents. The washed tissues were subsequently cut into 100‐µm sections using a vibratome. Tissue samples stored in PBS at 4°C served as untreated controls. All sections were washed three times with PBST (10 min each) and incubated with primary antibodies against NeuN and Iba1 at 4°C for 24 h. Following three additional washes in PBST (10 min each), sections were incubated with corresponding fluorophore‐conjugated secondary antibodies at room temperature for 2 h. The stained sections were then imaged under identical laser and acquisition settings using a spinning‐disk confocal microscope, and the resulting immunostaining fluorescence intensities were quantified using ImageJ.

### 4.10 Validation of Orthogonal Synergy and Optimization of Reagent Composition

1. Validation of hydrogen‐bond modulator necessity (Urea titration): To assess the contribution of the hydrogen‐bond modulation component to clearing performance, we tested the SynAtlas formulation with varying urea concentrations (0%–24%). The 0% urea condition served as a binary control (containing only the amphiphile and RI agent) to strictly validate the requirement for the third synergistic component. Brains were first incubated in delipidation–hydration solutions containing 10% 1,2‐hexanediol and varying concentrations of urea (0%–24%) at 37°C for 24 h. Samples were then transferred into RI‐matching solutions containing 10% 1,2‐hexanediol, 60% iohexol, and the corresponding urea concentration at 37°C for 24 h. Transparency was evaluated by bright‐field imaging.
2. Selection of the amphiphilic module (Comparison of surfactants): To validate the hypothesis that small‐molecule hydrotropes penetrate dense tissue matrices more effectively than micelle‐forming detergents, we compared the clearing efficiency of 1,2‐hexanediol against traditional surfactants. Brain hemispheres were incubated in delipidation–hydration solutions containing 24% urea and one of the following surfactants (each at 10%): 1,2‐hexanediol, sodium dodecyl sulfate (SDS), sodium dodecylbenzenesulfonate (SDBS), Tween 80, or Triton X‐100, at 37°C for 12 h. Samples were subsequently transferred into RI‐matching solutions containing 24% urea, 60% iohexol, and the same surfactant at 37°C for 12 h. Tissue transparency was assessed by bright‐field imaging.
3. Evaluation of high‐RI module compatibility: To assess the modular interchangeability of the high‐RI component and establish physicochemical compatibility criteria, we screened various high‐RI compounds within the orthogonal framework. Delipidation–hydration solutions containing 24% urea and 10% 1,2‐hexanediol were prepared. Brain hemispheres were incubated in these solutions at 37°C for 12 h, followed by transfer into RI‐matching solutions containing 24% urea, 10% 1,2‐hexanediol, and one of the high‐RI compounds (iohexol, antipyrine, 1‐(2‐hydroxyethyl)piperazine, uridine, 1,3‐dimethyl‐5‐pyrazolone, 3,6‐dithia‐1,8‐octanediol, nicotinamide, or creatinine) at 37°C for 12 h. Transparency and critical physicochemical compatibility (e.g., solubility in the high‐urea environment) were evaluated. Insoluble compounds were excluded from further analysis.
4. Validation of hydrogen‐bond modulator interchangeability: To assess the modular interchangeability of the hydrogen‐bond modulator, we evaluated N‐methyl‐D‐glucamine (NMDG) as an alternative to urea. Delipidation–hydration solutions containing 24% NMDG and 10% 1,2‐hexanediol were prepared. Brain hemispheres were incubated in these solutions at 37°C for 12 h, followed by transfer into RI‐matching solutions containing 24% NMDG, 10% 1,2‐hexanediol, and 60% iohexol at 37°C for 12 h. Tissue transparency was subsequently assessed by bright‐field imaging to verify whether alternative hydrogen‐bond modulators could successfully sustain the orthogonal synergy framework.
5. Evaluation of High‐RI Mixtures as Modular Components: To demonstrate that the orthogonal synergy principle guides functional modularity rather than rigid, single‐reagent combinations, we evaluated high‐RI binary mixtures as alternatives to monolithic high‐RI compounds across different hydrogen‐bond modulation environments. Delipidation–hydration solutions were prepared containing 10% 1,2‐hexanediol combined with either 24% urea, 24% NMDG, or 12% NMDG. Brain hemispheres were incubated in these initial solutions at 37°C for 12 h. Samples were subsequently transferred into corresponding RI‐matching solutions containing the identical hydrogen‐bond modulator profiles (24% urea, 24% NMDG, or 12% NMDG), 10% 1,2‐hexanediol, and one of the target high‐RI mixtures: the antipyrine/nicotinamide combination or the ionic liquid combination of 1‐(3‐aminopropyl)imidazole (NAPID) and phthalic acid (PA). Following incubation at 37°C for 12 h, macroscopic tissue transparency and critical physicochemical compatibility (such as room‐temperature solubility and crystallization resistance) were systematically evaluated.

Unless otherwise noted, all reagent concentrations refer to the final concentration in the solution; percentages are expressed as weight/volume (w/v) for solid reagents and volume/volume (v/v) for liquid reagents.

### 4.11 Optical Transmittance Measurements

To quantitatively evaluate optical transparency across different experimental conditions, spectral transmittance was recorded across the wavelength range of 400–800 nm. For the evaluation of individual SynAtlas components, 300‐µm‐thick brain slices were measured using a microplate reader (SpectraMax iD3, Molecular Devices). For benchmarking against comparative tissue‐clearing protocols and evaluating modular SynAtlas formulations, intact mouse hemibrains were assessed using a UV–vis–NIR spectrophotometer (UV‐3600 Plus, Shimadzu).

### 4.12 Assessment of Cryogenic Vitrification Capabilities and Microstructural Preservation

To evaluate the vitrification‐like properties and cryoprotective capacity of SynAtlas, two sets of experiments were performed. First, to assess the bulk phase behavior of the solutions, 5 mL centrifuge tubes containing 2 mL of either PBS or SynAtlas were immersed in liquid nitrogen for 3 min to achieve rapid freezing. Second, to assess tissue behavior, PFA‐fixed mouse brain hemispheres equilibrated in PBS and SynAtlas‐cleared hemispheres were subjected to the same rapid freezing protocol (liquid nitrogen for 3 min). All samples were subsequently transferred to a −80°C freezer and photographed immediately to assess macroscopic transparency and monitor for opacity or phase separation indicative of lattice ice crystallization. After thawing at room temperature, the brain hemispheres were sectioned into 100 µm slices using a vibratome. Control samples consisted of brain hemispheres that had not undergone freezing. Sections were imaged by confocal microscopy using a 405 nm excitation laser to visualize tissue architecture via autofluorescence. Quantitative analysis of microscopic tissue voids (pore size) was performed using ImageJ, serving as a proxy for evaluating structural disruption caused by ice crystal expansion. Third, to evaluate the preservation efficacy of fluorescent proteins and nucleic acids under long‐term low‐temperature storage, brain hemispheres from Thy1‐EYFP mice were first cleared using the SynAtlas protocol and then sectioned into left and right hemispheres. These paired hemispheres were stored at 4°C and −80°C for two weeks, respectively, followed by whole‐organ light‐sheet microscopy imaging. After intact imaging, the hemispheres were sectioned into 100 um slices using a vibratome to perform YFP fluorescence intensity quantification, PI staining, and FISH assays. Fourth, to evaluate the cryogenic properties and transparency maintenance of formulations incorporating alternative high‐RI modules (e.g., the antipyrine/nicotinamide mixture or the NAPID/PA ionic liquid), 5 mL centrifuge tubes containing 2 mL of either PBS or formulation variants (S1, S2, and S5) were placed directly into a −80°C freezer. After 24 h of incubation, the tubes were immediately photographed to assess their macroscopic transparency and verify their resistance to crystallization under low‐temperature storage.

### 4.13 Virus Injection

Two adeno‐associated viral (AAV) vectors were used for neural tracing: AAV‐flex‐mtdTomato and AAV/retro‐hSyn‐EGFP. AAV‐flex‐mtdTomato was injected into the PVN of 8–9‐week‐old CRH‐Cre mice (coordinates relative to bregma: AP −0.8 mm, ML −0.25 mm, DV −4.7 mm). AAV/retro‐hsyn‐EGFP was injected into the VTA of C57BL/6J mice (AP −3.08 mm, ML −0.55 mm, DV −4.24 mm). Mice were anesthetized with isoflurane and positioned in a stereotaxic apparatus. After leveling the cranial surface, a small craniotomy was made above the target site. Viral solutions were delivered through a glass micropipette connected to a microinjection pump at a rate of 50 nL/min, with an injection volume of 200 nL per site. Following infusion, the pipette was left in place for 5–10 min to minimize reflux before being slowly withdrawn. After surgery, mice were placed in a temperature‐controlled recovery chamber and returned to their home cages upon full recovery.

### 4.14 Nuclear Labeling with PI

Mouse whole brains and 1‐mm‐thick human brain sections fixed with 4% (w/v) PFA were washed in PBS three times (1 h each for whole brains, 30 min each for sections). Samples were then incubated in a 50% tetrahydrofuran/50% PBS solution containing PI (1:1000) at room temperature with gentle shaking (80 rpm) in the dark. For mouse whole brains, staining was performed for 3 days; for 1‐mm human brain sections, staining lasted for 1 day. After staining, samples were washed in PBS three times (1 h each for whole brains, 30 min each for sections). Subsequently, whole mouse brains were incubated in SynAtlas‐Delipidation solution at 37°C with gentle shaking (80 rpm) for 24 h, followed by clearing in SynAtlas solution at 37°C for an additional 24 h. In contrast, 1‐mm human brain sections were directly cleared in SynAtlas solution at 37°C for 24 h without a delipidation step.

### 4.15 Vascular Labeling with Lectin‐594

Mice were anesthetized with 0.8% sodium pentobarbital and positioned for intravenous injection. A total of 0.1 mL lectin‐594 was administered via the tail vein using a 1‐mL syringe. Immediately after injection, mice were transcardially perfused with 20 mL PBS followed by 20 mL of 4% PFA using a peristaltic pump. The brains and kidneys were then dissected, post‐fixed for subsequent processing.

### 4.16 Immunostaining of Mouse and Human Tissues

All samples were initially treated with SynAtlas‐Decolorization solution at 37°C with gentle shaking for 24 h to remove lipids and enhance tissue permeability to antibodies. Tissues were then washed three times with PBS (3 h each for whole tissues, 10 min each for sections) to remove residual SynAtlas‐Decolorization solution.

For whole mouse organs, tissues were incubated with the primary antibody (anti‐TH) diluted in 0.3% PBST containing 0.05% sodium azide at 32°C for 3 days with gentle shaking. After three washes in 0.3% PBST (3 h each), tissues were incubated with the corresponding secondary antibody solution at 32°C for 3 days, followed by another three PBST washes (3 h each). After immunostaining, mouse organs were cleared sequentially in SynAtlas‐Delipidation solution at 37°C with shaking (80 rpm) for 24 h, followed by clearing in SynAtlas solution at 37°C for an additional 24 h before imaging. For post‐clearing 2D immunostaining verification, the 3D‐stained kidney was sectioned into 100 µm slices using a vibratome. These sections were then incubated with the primary antibody (anti‐TH) diluted in 0.3% PBST containing 0.05% sodium azide at 4°C for 24 h. Following three washes in 0.3% PBST (5 min each), the sections were incubated with the corresponding secondary antibody solution at room temperature (RT) for 2 h.

For 300 µm human brain sections, after PBS washes, sections were incubated with the primary antibody diluted in 0.3% PBST at 32°C overnight, washed three times in PBS (10 min each), and then incubated with the secondary antibody at 32°C overnight.

Subsequently, the sections were washed three times in PBS (10 min each). For lectin and DAPI labeling, sections were incubated in a staining solution containing lectin‐594 (1:500) and DAPI (1:1000) for 6 h at room temperature.

For multi‐round staining, after the first round of labeling, tissues were incubated in an immunostain stripping buffer containing 4 M guanidine hydrochloride and 2% β‐mercaptoethanol at 45°C for 2 h to disrupt antigen‐antibody interactions and remove the previous antibody signals. After stripping, the sections were washed three times in PBS (10 min each) and subjected to the second round of immunostaining following the same procedure as in the first round. After three additional PBS washes, sections were directly cleared in SynAtlas at room temperature for 10 min before imaging.

To evaluate the effect of the stripping buffer treatment on antigen preservation, serial human brain sections were initially treated with SynAtlas‐Decolorization solution at 37°C with gentle shaking for 24 h to remove lipids. The sections were then randomly allocated into two parallel groups: one group was treated with the stripping buffer at 45°C for 24 h, while the control group was incubated in PBS at 45°C for 24 h. Subsequently, both groups were immunostained for GFAP under identical conditions as described above. Following immunostaining, the sections were imaged using a spinning‐disk confocal microscope under identical laser and acquisition parameters, and the resulting fluorescence signal intensities were quantified using ImageJ. The antibodies used are listed in Table S3.

### 4.17 Fluorescence In Situ Hybridization of Whole Heart Tissue

Whole hearts were fixed in 4% (w/v) PFA in PBS for 24 h at 4°C and subsequently treated with SynAtlas‐Decolorization solution at 37°C for 4 h to remove heme pigments and enhance tissue permeability. Multiplexed FISH targeting *Myl2* and *Nppa* was performed using HCR v3.0 technology. Following decolorization, tissues were washed three times with PBS (1 h each) to thoroughly remove residual clearing reagents, and then pre‐hybridized in hybridization buffer (30% formamide, 5× SSC, 0.1% Tween‐20) at 37°C for 1 h. Tissues were then incubated in hybridization buffer containing split‐initiator probe sets against *Myl2* and *Nppa* at 37°C for 24 h. Subsequently, samples were washed three times in hybridization buffer (1 h each, 37°C), followed by a single wash in 5× SSC containing 0.1% Tween‐20 (SSCT) for 1 h at room temperature. For signal amplification, fluorescently labeled hairpins (B1‐Alexa647 and B2‐Alexa488) were snap‐cooled and incubated with the samples in 5× SSCT at room temperature for 24 h in the dark. After hairpin amplification, samples were washed three times in 5× SSCT (1 h each, room temperature). Tissues were cleared in SynAtlas solution at room temperature for 24 h prior to volumetric imaging using a light‐sheet fluorescence microscope.

### 4.18 Multi‐Round and Multiplex In Situ Hybridization of Whole Pituitary Tissue

Whole pituitaries were fixed in 4% (w/v) PFA for 24 h at 4°C and subsequently delipidated in SynAtlas‐Delipidation solution supplemented with 1% RNase inhibitor polyvinyl sulfonic acid (PVSA) at 37°C for 24 h. Multiplexed FISH was performed using HCR v3.0 technology [43]. For the first round of hybridization, split‐initiator probe sets against *Tshb*, *Prl*, and *Lhb* were applied in hybridization buffer (30% formamide, 5× SSC, 0.1% Tween‐20, 1% PVSA) and incubated at 37°C for 24 h. Samples were then washed three times in hybridization buffer (3 h each, 37°C), followed by a single wash in 5× SSC/0.1% Tween‐20 for 3 h. Fluorescent hairpins (B1‐Alexa647, B2‐Alexa488 and B3‐Alexa555) were hybridized in 5× SSC/0.1% Tween‐20/1% PVSA at room temperature for 24 h in the dark. After hybridization, samples were washed three times in 5× SSC / 0.1% Tween‐20 (3 h each, room temperature). Nuclei were counterstained with DAPI (1 µg/ml in PBS containing 1% PVSA) for 24 h in the dark. Tissues were cleared in SynAtlas solution at room temperature for 1 h prior to imaging with a spinning‐disk confocal microscope. After image acquisition, tissues were washed twice with PBS (1 h each) to remove residual SynAtlas reagents. Probes and hairpins were stripped by incubating tissues in stripping buffer (60% formamide, 0.5× SSC, 1% SDS) at 55°C for 2 h. A second round of hybridization was then conducted using probes against *Pomc*, *Gh*, and *Fshb*, following the same procedure described above, including DAPI counterstaining, tissue clearing, and imaging. This workflow enabled multi‐round, multiplex FISH of intact pituitary tissue. The specific HCR probes utilized in this study are listed in Table S4.

### 4.19 Imaging and 3D Reconstruction of Cleared Samples

Cleared whole‐mount samples were imaged using a Light‐Sheet Microscope (LCS SPIM, Luxendo‐Bruker, Heidelberg, Germany), equipped with a 4.0× objective lens (Olympus XLFLUOR 4x/340, NA = 0.28). The system employed combined laser lines at 488 nm, 561 nm, and 642 nm, each with an input power of 40 mW, and appropriate emission filters. Images were acquired with effective magnifications of 4.4× or 8.9× using LuxControl 4.2.2 software.

Tissue slices were imaged using a Spinning Disk Confocal Microscope (SpinSR, Olympus), equipped with the following objectives: 5× (NA = 0.25, WD = 12.5 mm), 10× (NA = 0.5, WD = 2 mm), 20× (NA = 0.8, WD = 0.55 mm), and 60× (NA = 1.4, WD = 0.13 mm).

All light‐sheet datasets were processed to generate image stacks. For datasets requiring depth visualization, depth‐based color coding was performed using the ImageJ (Fiji, version 1.54p) ZstackDepthColorCode (v.0.0.2) plugin, which maps the *z*‐axis position of each voxel to a color gradient. Subsequent 3D reconstructions were then performed in Imaris (Bitplane, version 9.3). For datasets not requiring depth coding, 3D reconstructions were carried out directly in Imaris for visualization. All 3D images were rendered and exported for figure preparation.

### 4.20 Whole‐Brain Registration and Cell Quantification

Whole‐brain datasets from ChAT‐Cre:Ai6 mice were registered to the Allen Common Coordinate Framework version 3 (CCFv3) using the BIRDS pipeline [44, 45], following procedures described in the original publication. After registration, datasets were masked according to Allen CCFv3 regional annotations to extract 3D imaging data of ChAT‐Cre:Ai6–labeled cells in each brain region. These region‐specific datasets were imported into Imaris, where the Spot function was executed via Python XTension for automated cell detection and quantification. Regional cell counts were compiled and exported for further analysis. Abbreviations and full names of major brain regions are listed in Table S2.

### 4.21 3D Registration of Multi‐Round Mouse Pituitary FISH and Human Brain Slice Immunostaining Datasets

For multi‐round, multiplexed in situ hybridization datasets of whole pituitary tissue, image registration was performed using BigStream software [38] with the DAPI channel serving as the reference. Image stacks from both hybridization rounds were converted into Zarr format and processed through the BigStream pipeline. A Difference of Gaussian (DoG) filter was applied to 4× downsampled image pairs (4 × 0.864 × 0.864 µm), and local maxima above a predefined threshold were extracted as features. These features were matched using RANSAC, followed by global alignment via affine transformation. The raw datasets were then partitioned into overlapping chunks (256 × 512 × 512 pixels, with 50% overlap) for local refinement. On each chunk, feature detection and affine alignment were performed at full resolution (1 × 0.217 × 0.217 µm), followed by deformable registration to generate a dense displacement field. This deformation field was applied to all channels of the second‐round dataset, and the transformed volumes were integrated with the first‐round images. To assess registration accuracy, the Structural Similarity Index (SSIM) was computed between the DAPI channel of the second‐round dataset after registration and the corresponding DAPI channel of the first‐round dataset, providing a quantitative measure of spatial alignment fidelity. Similarly, for the two‐round human brain slice immunostaining datasets, 3D registration was also performed using the BigStream pipeline, with the lectin‐594 channel from both rounds serving as the reference for alignment.

### 4.22 Segmentation of Pituitary Hormone‐Producing Cells

Automatic segmentation of pituitary cells labeled with DAPI, *Pomc*, *Gh*, *Lhb*, and *Tshb* was performed using Cellpose 3 with the cyto3 model [46]. Images were sampled along the dorsoventral axis of the pituitary at 15‐µm intervals, and each optical plane was processed to segment individual cells. For certain hormone markers such as *Tshb*, where cells exhibited tight clustering, the cyto3 model occasionally merged adjacent cells into a single segmentation mask. To improve accuracy, segmentation results from the DAPI channel were used as a spatial reference to calibrate hormone‐channel segmentation. Specifically, the centroid of each DAPI‐derived mask was taken as the centroid of a single cell, minimizing segmentation errors in densely packed regions. All segmentation results were manually inspected to ensure accuracy.

### 4.23 Statistical Analysis

All quantitative analyses were performed using GraphPad Prism v9.0.0 software. Data are presented as mean ± standard error of the mean (SEM) unless otherwise indicated, and the number of biological replicates (n) is specified in the corresponding figure legends. For comparisons between two groups, two‐tailed Student's *t*‐tests were used, with paired t‐tests applied to matched measurements from the same specimen, such as tissue dimensions before and after clearing, and unpaired *t*‐tests applied to independent experimental groups. Comparisons among three or more groups were performed using one‐way analysis of variance (ANOVA) followed by Bonferroni's multiple‐comparison test. A *p* value < 0.05 was considered statistically significant. Fluorescence signal intensity, background fluorescence, tissue dimensions, and microscopic pore size were quantified using ImageJ/Fiji under identical analysis settings across experimental groups.

## Author Contributions

**Mei‐Xue Yuan**: investigation, methodology, data curation, formal analysis, writing – review and editing, writing – original draft, validation, visualization. **Xin‐Ya Qin**: conceptualization, investigation, funding acquisition, methodology, formal analysis, data curation, writing – review and editing, writing – original draft, validation, resources. **Ling‐Jie Li**: methodology, investigation. **Kai‐Lang Xiao**: methodology, investigation. **Meng‐Meng Zhang**: methodology, investigation. **Kun‐Qi Chen**: methodology, investigation. **Peng Chen**: methodology, software, resources. **Yu Wang**: methodology, software, resources. **Chen‐Wei Wang**: methodology, software, resources. **Nan Zhou**: supervision, resources, funding acquisition, investigation, writing – original draft, conceptualization, writing – review and editing, visualization, project administration. **Qing‐Hong Shan**: conceptualization, investigation, funding acquisition, writing – original draft, writing – review and editing, visualization, validation, methodology, formal analysis, project administration, resources, supervision, data curation.

## Funding

This work was supported by the STI2030‐Major Projects (grant no. 2022ZD0205202), the National Natural Science Foundation of China (grant no. 32200798, 82471483 and 82471540), the University Synergy Innovation Program of Anhui Province (grant no. GXXT‐2022‐030), and the Key Natural Science Research Projects in Anhui Province's Universities (grant no. 2024AH050821).

## Conflicts of Interest

Q.‐H.S., X.‐Y.Q., N.Z., and M.‐X.Y. submitted patent applications related to the SynAtlas method developed in this work. The other authors declare no competing interests.

## Supporting information




**Supporting File 1**: advs78739‐sup‐0001‐SuppMat.docx.

**Supporting File 2**: advs78739‐sup‐0002‐VideoS1.mp4.

**Supporting File 3**: advs78739‐sup‐0003‐VideoS2.mp4.

**Supporting File 4**: advs78739‐sup‐0004‐VideoS3.mp4.

**Supporting File 5**: advs78739‐sup‐0005‐VideoS4.mp4.

**Supporting File 6**: advs78739‐sup‐0006‐VideoS5.mp4.

**Supporting File 7**: advs78739‐sup‐0007‐VideoS6.mp4.

**Supporting File 8**: advs78739‐sup‐0008‐VideoS7.mp4.

**Supporting File 9**: advs78739‐sup‐0009‐VideoS8.mp4.

**Supporting File 10**: advs78739‐sup‐0010‐VideoS9.mp4.

**Supporting File 11**: advs78739‐sup‐0011‐dataS1.xlsx.

**Supporting File 12**: advs78739‐sup‐0012‐dataS2.xlsx.

**Supporting File 13**: advs78739‐sup‐0013‐dataS3.xlsx.

**Supporting File 14**: advs78739‐sup‐0014‐dataS4.xlsx.

**Supporting File 15**: advs78739‐sup‐0015‐dataS5.xlsx.

## Data Availability

The data that support the findings of this study are available from the corresponding author upon reasonable request.
