# Supplementary material for: SynAtlas: A Safe and Versatile Platform for Rapid Organ‐Scale Tissue Clearing and 3D Atlas Mapping
Source: Adv Sci (Weinh). 2026 Nov 9:e78739. Online ahead of print. doi: 10.1002/advs.78739 (PMC13748908; doi:10.1002/advs.78739)
Supplement: Supplementary file 1 [file ADVS-9999-e78739-s010.docx]

Supporting Information

Title

SynAtlas: A Safe and Versatile Platform for Rapid Organ-Scale Tissue Clearing and 3D Atlas Mapping

Author(s), and Corresponding Author(s)

*Mei-Xue Yuan, Xin-Ya Qin^*^, Ling-Jie Li, Kai-Lang Xiao, Meng-Meng Zhang, Kun-Qi Chen, Peng Chen, Yu Wang, Chen-Wei Wang, Nan Zhou^*^, Qing-Hong Shan^*^*

**This file includes:**

Figures S1 to S11

Tables S1 to S5

Legends for Movies S1 to S9

**Other supporting information for this manuscript include the following:**

Movies S1 to S9

**Figure S1. Screening workflow and functional categorization of reagents for SynAtlas development**

(A) Workflow of SynAtlas reagent screening from 1,631 hydrophilic candidates derived from CUBIC reagent libraries.

(B) Chemical structures and GHS signal words of representative hydration-promoting reagents.

(C) Chemical structures and GHS signal words of representative delipidation reagents.

(D) Chemical structures and GHS signal words of representative high–RI reagents.

**Figure S2. Tissue clearing performance and dimensional stability of SynAtlas in thick brain sections.**

(A) Representative clearing results of brain sections with different thicknesses (500, 1000, and 2000 µm) at varying treatment times. Scale bar, 2.5 mm.

(B) Quantification of linear tissue expansion after 24-h SynAtlas treatment (n = 3, mean ± SEM; ns: not significant by paired t-test).

(C-D) Clearing of intact mouse brains using SynAtlas. Single-step SynAtlas treatment (C); lower two-step protocol (SynAtlas-Delipidation followed by SynAtlas) (D). Scale bars, 2.5 mm.

**Figure S3. Comparative evaluation of nucleic acid preservation and immunostaining quality across different tissue-clearing methods**

(A) Representative confocal images of brain sections showing total nucleic acid preservation via PI and DAPI staining across PBS control, SynAtlas, EZ Clear, OptiMuS, CUBIC, CUBIC-L/R, and VIVIT treatment groups. Scale bar, 50 μm.

(B) Quantification of relative PI fluorescence intensities in tissues processed by different clearing methods compared to PBS-treated controls (n = 3 for each group).

(C) Representative multiplexed FISH images targeting Sst and Vglut1 mRNA transcripts using HCR FISH across the indicated tissue clearing groups, highlighting mRNA signal retention or degradation. Scale bar, 100 μm.

(D) Representative immunofluorescence images of NeuN (green) and Iba1 (magenta) staining in mouse brain sections following treatment with PBS, SynAtlas, EZ Clear, OptiMuS, CUBIC, CUBIC-L/R, or VIVIT. Scale bar, 100 µm.

(E–G) Quantification of NeuN fluorescence signal intensity (E), background fluorescence intensity (F), and signal-to-noise ratio (SNR) (G) (n = 3 for each group).

(H–J) Quantification of Iba1 fluorescence signal intensity (H), background fluorescence intensity (I), and SNR (J) (n = 3 for each group).

**Figure S4. Evaluation of long-term low-temperature storage on fluorescent protein and nucleic acid biomarker preservation in SynAtlas-cleared tissues.**

(A) Schematic workflow illustrating the two-week storage evaluation protocol of SynAtlas-cleared Thy1-EYFP mouse brain hemispheres at either 4 ℃ or -80 ℃.

(B) Representative whole-organ light-sheet microscopy imaging of SynAtlas-cleared brain hemispheres after two weeks of storage at 4 ℃ or -80 ℃, showing the preservation of endogenous YFP signals. Scale bar, 2 mm.

(C) Representative confocal images of tissue sections from SynAtlas-cleared hemispheres, prepared after a two-week storage period at 4 ℃ or -80 ℃, showcasing endogenous YFP signal retention. Scale bar, 50 μm.

(D) Quantitative analysis of YFP sectional signal density comparing the 4 ℃ and -80 ℃ storage groups (n = 8 for each group).

(E) Representative confocal images of tissue sections stained with PI to visualize total RNA preservation following two weeks of storage under the indicated temperature conditions. Scale bar, 50 μm.

(F) Quantitative analysis of relative PI staining fluorescence intensities between the 4 ℃ and -80 ℃ storage groups (n = 6 for each group).

(G) Representative multiplexed HCR FISH images showing robust mRNA labeling performance in SynAtlas-processed tissues after two weeks of storage at either 4 ℃ or -80 ℃. Scale bar, 100 μm.

**Figure S5. Functional validation and optimization of the orthogonal synergy components in SynAtlas clearing**

(A) Workflow for testing the effects of varying urea concentrations on tissue transparency.

(B) Representative clearing results of whole mouse brains treated with 60% (w/v) iohexol and 10% (v/v) 1,2-hexanediol combined with different urea concentrations (0, 6, 12, 18, and 24% (w/v)). Only 24% (w/v) urea achieved complete transparency. Scale bar, 2.5 mm.

(C) Workflow for comparing different delipidation/amphiphilic agents on tissue transparency.

(D) Representative clearing results obtained with various delipidation reagents (1,2-hexanediol vs. SDS, SDBS, Tween-80, and Triton X-100) in combination with 24% (w/v) urea and 60% (w/v) iohexol. 1,2-hexanediol showed superior overall transparency. Scale bar, 2.5 mm.

(E) Workflow for testing different high-refractive-index (high-RI) agents on tissue transparency.

(F) Representative clearing results of soluble high-RI reagents (e.g., iohexol, antipyrine, uridine) in solutions containing 24% (w/v) urea and 10% (v/v) 1,2-hexanediol. Scale bar, 2.5 mm.

(G) Spectral transmittance of representative SynAtlas formulations containing different amphiphilic or high-RI components, measured across 400–800 nm. (n = 3 per group).

(H) Solubility test results for high-RI reagents (e.g., Nicotinamide, Creatinine) in 24% (w/v) urea and 10% (v/v) 1,2-hexanediol solutions, showing insolubility in some cases.

**Figure S6. Validation of the orthogonal synergy principle through modular component substitution and clearing efficacy assessment.**

(A) Schematic workflow illustrating the modular substitution testing protocol utilizing NMDG as an alternative hydrogen-bond modulator combined with 1,2-hexanediol and iohexol.

(B-C) Chemical structure of NMDG (B) and representative macroscopic image of the grid background through a cleared sample (C). Scale bar, 2.5 mm.

(D) Chemical structures of alternative high-RI modules evaluated for refractive index matching, including PA, NAPID, antipyrine, and nicotinamide.

(E) Formulations and detailed chemical compositions of the screening variants designated S1, S2, S3, S4, and S5.

(F) Representative photograph illustrating the room-temperature physical state and solution stability of formulation variants S1 to S5, highlighting the precipitation/crystallization behaviors in S3 and S4 versus the stable liquid phases in S1, S2, and S5.

(G) Schematic workflow illustrating the generalized two-step clearing evaluation protocol using combinations of distinct hydrogen-bond modulators (X) and high-RI matching modules (Y).

(H) Representative macroscopic images of mouse half brains before (PBS control) and after tissue clearing with the stable formulation variants S1, S2, and S5. Scale bar, 2.5 mm.

(I-J) Representative photographs comparing the physical states of PBS control and formulations S1, S2, and S5 stored at room temperature (I) and after cryogenic exposure at -80 ℃ for 24 h (J), demonstrating the distinct crystallization resistance and transparency retention of S2 and S5 under low-temperature storage.

**Figure S7. Screening and optimization of decolorization module for heme-rich organs**

(A) Schematic workflow for screening decolorizing agents and optimizing their concentrations in the SynAtlas-Delipidation formulation. Kidney and spleen tissues were incubated for 24 h at 37 °C in solutions containing 10% (v/v) 1,2-hexanediol, 24% (w/v) urea, and either N-methyldiethanolamine, N-butyldiethanolamine, or Quadrol at 10% or 20% (v/v).

(B) Evaluation of decolorization efficiency in kidney and spleen tissues treated with SynAtlas-Delipidation (24% (w/v) urea and 10% (v/v) 1,2-hexanediol) combined with different decolorizing agents or concentrations (Quadrol, N-butyldiethanolamine, N-methyldiethanolamine). N-butyldiethanolamine showed the strongest synergistic effect. Scale bar, 2.5 mm.

(C) Schematic workflow for evaluating Quadrol and N-methyldiethanolamine as alternative decolorizing agents. Intact organs were first incubated for 24 h at 37 °C in a solution containing 10% (v/v) 1,2-hexanediol, 24% (w/v) urea, and either 20% (v/v) Quadrol or 20% (v/v) N-methyldiethanolamine, followed by incubation in SynAtlas for an additional 24 h at 37 °C.

(D) Representative images of heart, kidney, liver, and spleen after treatment with SynAtlas-Delipidation alone or with the indicated decolorizing agents, followed by SynAtlas clearing. Both Quadrol and N-methyldiethanolamine markedly improved decolorization and tissue transparency in heme-rich organs, demonstrating their suitability as alternative reagents for the SynAtlas decolorization module. Scale bar, 2.5 mm.

**Figure S8. 3D mapping of diverse fluorescent protein labels and automated cell detection in SynAtlas-cleared mouse brains**

(A) Representative whole-brain light-sheet microscopy image of a cleared Thy1-YFP mouse brain (A) and high-magnification views of endogenous YFP-labeled neurons and projections in the cortex (a1), hippocampal formation (HPF) (a2), and basolateral amygdala (BLA) (a3). Scale bars: (A), 1 mm; (a1–a3), 100 μm.

(B) Representative whole-brain image of a cleared CRH-Cre;Ai9 mouse brain (B) and high-magnification views of endogenous tdTomato signals in the cortex (b1), diagonal band of Broca (NDB) (b2), and paraventricular nucleus of the hypothalamus (PVN) (b3). Scale bars: (B), 1 mm; (b1–b3), 100 μm.

(C) Representative whole-brain image of a cleared VIP-Cre;Ai6 mouse brain (C) and high-magnification views of endogenous ZsGreen signals in the cortex (c1), entorhinal area (ENT) (c2), and olfactory bulb (OB) (c3). Scale bars: (C), 1 mm; (c1–c3), 100 μm.

(D) Representative whole-brain image of a cleared ChAT-Cre;Ai6 mouse brain (D) and high-magnification views of choline acetyltransferase (ChAT) neurons in the cortex (d1), medial septal nucleus (MS) (d2), and hippocampal formation (HPF) (d3). Scale bars: (D), 1 mm; (d1–d3), 100 μm.

(E) Representative whole-brain horizontal views showing endogenous ZsGreen signals in a ChAT-Cre;Ai6 mouse brain alongside the corresponding automatically detected cell spots color-coded by anterior-to-posterior position (left). High-magnification panels (right) demonstrate the fidelity of automated cell identification in Imaris by overlaying original GFP channels (green) with automated spot segmentations (blue) at two different magnifications. Scale bars: (left panels), 1 mm; (right panels), 100 μm and 50 μm.

**Figure S9. Validation of deep-tissue 3D molecular imaging in mouse kidney and heart using SynAtlas**

(A-B) Representative cross-sectional views from two independent mouse kidney samples (A and B) showing the spatial distribution of sympathetic fibers labeled via 3D whole-tissue immunostaining (3D: anti-TH, magenta) overlaid with the signal obtained from post-clearing 2D sectioning and immunostaining (Post 2D: anti-TH, green). Dashed boxes indicate the regions selected for high-magnification analysis in the renal cortex (a1, b1) and near the deep medullary/vasculature areas (a2, b2). Individual channels and merged views demonstrate the precise alignment and high fidelity of the 3D-rendered sympathetic network against traditional 2D section labeling. Scale bars: (A, B), 1 mm; (a1–b2 panels), 50 μm.

(C) Whole-mount 3D reconstruction of an intact mouse heart immunostained for tyrosine hydroxylase (TH) following SynAtlas-Decolorization and SynAtlas clearing, showing extensive sympathetic nerve networks throughout the cardiac tissue. Blue boxes indicate the regions shown at higher magnification in (D) and (E). Scale bar, 500 µm.

(D–E) Magnified views of the boxed regions in (C), showing TH-positive sympathetic fibers across the epicardial surface and within the deep myocardium, including fine branching nerve fibers. Scale bars, 200 µm.

(F–H) Whole-mount HCR-FISH of *Myl2* and *Nppa* in an intact mouse heart after SynAtlas processing. (F) Merged 3D reconstruction showing *Myl2* (magenta) and *Nppa* (green) expression. (G) *Myl2* signal showing predominant enrichment in the ventricular compartment. (H) *Nppa* signal showing predominant enrichment in the atrial compartments. Scale bars, 500 µm.

**Figure S10. Validation of registration and segmentation for multiplexed pituitary FISH**

(A to B) Comparison of DAPI channels from two rounds of multiplexed FISH staining in the same pituitary sample after registration. (A) Top view; (B) front view. Scale bars, 500 µm.

(C, E) Representative sectional views from (A), illustrating the global quality of image registration. Scale bars, 400 µm for (C); 500 µm for (E).

(D, F) Higher-magnification views of the boxed regions in (C) and (E), demonstrating single-cell-level alignment accuracy after registration. Scale bars in (D, F), 30 µm.

(G) Cumulative frequency plot showing the distribution of SSIM scores as a measure of overall registration performance.

(H) CellPose-based segmentation of pituitary cells across different fluorescence channels. Scale bar, 25 µm.

(I) Segmentation results of DAPI- and Fshb-labeled pituitary cells using CellPose. Scale bar, 20 µm.

(J) Example illustrating improved segmentation accuracy through DAPI-assisted boundary correction. Scale bar, 20 µm.

(K) Spatial mapping of *Fshb*-positive cells after DAPI-guided segmentation refinement. Scale bar, 20 µm.

Figure S11. Effect of stripping buffer treatment on GFAP antigenicity in human brain tissue.

(A) Schematic of the experimental workflow for evaluating antigen preservation following stripping. Serial human brain sections were subjected to SynAtlas decolorization and subsequently incubated in either PBS (non-stripped) or stripping buffer (stripped) at 45 °C for 24 h, followed by GFAP immunostaining and imaging under identical conditions.

(B) Representative GFAP immunofluorescence images of non-stripped and stripped sections after subsequent GFAP immunostaining. Scale bar, 100 µm.

(C) Quantification of GFAP mean fluorescence intensity in non-stripped and stripped sections. Data are presented as mean ± SEM (n = 3 for each group**).**

**Table S1. List of candidate reagents for SynAtlas screening and their toxicity profiles**

This table provides a comprehensive list of the reagents evaluated in this study. It includes chemical names and safety data, specifically Globally Harmonized System (GHS) hazard classifications and acute oral toxicity (LD50) values. These data were used to filter the initial library of 1,631 candidates down to 293 low-hazard reagents for further functional testing.

**Table S2. Abbreviations and nomenclature of brain regions for automated quantification**

List of anatomical regions and their corresponding abbreviations based on the Allen Mouse Brain Common Coordinate Framework version 3 (CCFv3), corresponding to the quantification data presented in Fig. 2.

**Table S3. List of primary and secondary antibodies used for immunostaining.**

Summary of antibodies used for mouse and human tissue labeling, including immunogen, host species, source (vendor and catalog number), and dilution ratios used in this study.

**Table S4. List of HCR FISH probes used for fluorescence in situ hybridization**

This table summarizes the specific split-initiator probe sets utilized for third-generation hybridization chain reaction (HCR v3.0) multiplexed fluorescence in situ hybridization (FISH), including *Vglut1, Sst, Tshb, Prl, Lhb, Gh, Fshb,* *Pomc, Myl2* and *Nppa*.

**Table S5. Comparative overview of representative tissue-clearing methods and SynAtlas**

This table summarizes the major features, advantages, limitations, and application compatibility of representative tissue-clearing methods and SynAtlas.

**Movie S1. Whole-brain registration and regional segmentation of cholinergic neurons in a ChAT-Cre;Ai6 mouse.**
This video demonstrates the result of registering a SynAtlas-cleared ChAT-Cre;Ai6 whole-brain dataset to the Allen Brain Atlas (CCFv3). It visualizes the precise anatomical segmentation and 3D distribution of Choline Acetyltransferase (ChAT) positive neurons across different brain regions, as quantified in Fig. 2.

**Movie S2. Three-dimensional visualization of anterograde projections from the paraventricular nucleus (PVN).**
A 3D fly-through of a SynAtlas-cleared CRH-Cre mouse brain injected with AAV-DIO-mtdTomato in the PVN. The video highlights intact long-range projections extending from the hypothalamus to distant targets, including the nucleus tractus solitarius (NTS) and entorhinal cortex.

**Movie S3. Whole-brain mapping of retrograde inputs to the ventral tegmental area (VTA).**
Visualization of a C57BL/6J mouse brain injected with AAV/retro-hSyn-EGFP in the VTA. The video reveals the 3D architecture of retrograde inputs, showing projecting neuronal somata and fibers in upstream regions such as the striatum and anterior cingulate cortex (ACC).

**Movie S4. Uniform whole-brain nuclear labeling and neuronal imaging using SynAtlas.**
Optical sectioning (Z-stack fly-through) of a Thy1-YFP mouse brain co-labeled with Propidium Iodide (PI). The video demonstrates the uniform penetration of the nuclear stain (red) throughout the deep brain regions while preserving the endogenous YFP fluorescence (green).

**Movie S5. High-resolution 3D reconstruction of the whole-mouse brain vascular network.**
A 3D rendering of a C57BL/6J mouse brain with vasculature labeled via intravenous injection of Lycopersicon esculentum (tomato) lectin. The video details the intact vascular hierarchy, from major vessels to fine capillaries in the brain.

**Movie S6. Volumetric imaging of renal vasculature and glomerular architecture.**
Three-dimensional visualization of a SynAtlas-cleared kidney labeled with Lectin-594. The video showcases the complex spatial organization of the renal vascular system, resolving fine capillary networks and glomeruli structures.

**Movie S7. Three-dimensional mapping of sympathetic innervation in the intact kidney.**
Visualization of whole-mount immunostaining against Tyrosine Hydroxylase (TH) in a mouse kidney. The video reveals the 3D architecture of the sympathetic nervous system, highlighting nerve fibers tracking along the renal vasculature and innervating the cortex and medulla.

**Movie S8. Spatial organization of six hormone-secreting cell types in the whole pituitary gland.**
A composite 3D reconstruction derived from two rounds of multiplexed FISH in a whole mouse pituitary. The video displays the spatial distribution of six distinct cell populations expressing *Tshb, Prl, Lhb, Pomc, Gh, and Fshb* mRNAs at single-cell resolution.

**Movie S9. Multi-scale imaging of a large tissue slab from an adult Thy1-YFP mouse.**
3D rendering of a 2-mm-thick coronal body slab from an adult mouse, cleared using the embedding-sectioning SynAtlas protocol. The video zooms in from a macroscopic view to resolve individual neuronal somata in the brain and peripheral nerve bundles in facial regions.
